# Thrombospondin 4/integrin α2/HSF1 axis promotes proliferation and cancer stem-like traits of gallbladder cancer by enhancing reciprocal crosstalk between cancer-associated fibroblasts and tumor cells

**DOI:** 10.1186/s13046-020-01812-7

**Published:** 2021-01-06

**Authors:** Yu Shi, Liankang Sun, Rui Zhang, Yuan Hu, Yinying Wu, Xuyuan Dong, Danfeng Dong, Chen Chen, Zhimin Geng, Enxiao Li, Yangwei Fan

**Affiliations:** 1 Department of Medical Oncology, The First Affiliated Hospital of Xi’an Jiaotong University, 277 Yanta West Road, Xi’an, 710061 Shaanxi Province China; 2 Department of Hepatobiliary Surgery, The First Affiliated Hospital of Xi’an Jiaotong University, 277 Yanta West Road, Xi’an, 710061 Shaanxi Province China

**Keywords:** Gallbladder cancer, Cancer-associated fibroblasts, Thrombospondin 4, Heat shock factor 1, Proliferation, Cancer stemness

## Abstract

**Background:**

Cancer-associated fibroblasts (CAFs), the primary component of tumor stroma in tumor microenvironments, are well-known contributors to the malignant progression of gallbladder cancer (GBC). Thrombospondins (THBSs or TSPs) comprise a family of five adhesive glycoproteins that are overexpressed in many types of cancers. However, the expression and potential roles of TSPs in the crosstalk between CAFs and GBC cells has remained unclear.

**Methods:**

Peritumoral fibroblasts (PTFs) and CAFs were extracted from GBC tissues. Thrombospondin expression in GBC was screened by RT-qPCR. MTT viability assay, colony formation, EdU incorporation assay, flow cytometry analysis, Transwell assay, tumorsphere formation and western blot assays were performed to investigate the effects of CAF-derived TSP-4 on GBC cell proliferation, EMT and cancer stem-like features. Subcutaneous tumor formation models were established by co-implanting CAFs and GBC cells or GBC cells overexpressing heat shock factor 1 (HSF1) to evaluate the roles of TSP-4 and HSF1 in vivo. To characterize the mechanism by which TSP-4 is involved in the crosstalk between CAFs and GBC cells, the levels of a variety of signaling molecules were detected by coimmunoprecipitation, immunofluorescence staining, and ELISA assays.

**Results:**

In the present study, we showed that TSP-4, as the stromal glycoprotein, is highly expressed in CAFs from GBC and that CAF-derived TSP-4 induces the proliferation, EMT and cancer stem-like features of GBC cells. Mechanistically, CAF-secreted TSP-4 binds to the transmembrane receptor integrin α2 on GBC cells to induce the phosphorylation of HSF1 at S326 and maintain the malignant phenotypes of GBC cells. Moreover, the TSP-4/integrin α2 axis-induced phosphorylation of HSF1 at S326 is mediated by Akt activation (p-Akt at S473) in GBC cells. In addition, activated HSF1 signaling increased the expression and paracrine signaling of TGF-β1 to induce the transdifferentiation of PTFs into CAFs, leading to their recruitment into GBC and increased TSP-4 expression in CAFs, thereby forming a positive feedback loop to drive the malignant progression of GBC.

**Conclusions:**

Our data indicate that a complex TSP-4/integrin α2/HSF1/TGF-β cascade mediates reciprocal interactions between GBC cells and CAFs, providing a promising therapeutic target for gallbladder cancer patients.

## Background

Gallbladder cancer (GBC) is the most common and fatal malignancy of the biliary tract [1]. Due to the lack of early symptoms, most patients with gallbladder cancer are diagnosed in advanced stages and may receive unsatisfactory treatments, resulting in a bleak prognosis [1]. The median survival time of GBC patients is less than 1 year [2]. Metastasis is the primary threat to the lives of GBC patients due to the lack of effective therapeutic options [3]. Thus, there is an urgent need to elucidate the molecular mechanism that drives GBC metastasis.

Growing evidence suggests that tumor metastasis is not only determined by epigenetic or genetic networks in cancer cells but is also influenced by the tumor microenvironments [4, 5]. Cancer-associated fibroblasts (CAFs), the main component of tumor stroma in tumor microenvironments, can build-up the cross-talk with cancer cells to facilitate tumor growth, metastasis, immunosuppression, and chemoresistance as well as the creation of a niche for maintaining cancer stemness [6–9]. In human lung cancer, the tumor microenvironment harbors abundant CAFs that secrete IGF-II, a signaling molecule that binds to IGF1R on lung CSCs to activate Akt/Nanog cascades, which play crucial roles in maintaining lung cancer stemness [10]. Moreover, the disruption of IGF-II/IGF1R signaling represses Nanog expression and restrains cancer stem-like features in lung cancer [10]. Analogously, via paracrine IL-1α/β signaling, disseminated breast cancer cells reeducate lung fibroblasts and induce their transdifferentiation into CAFs to generate a metastatic niche for breast cancer. Subsequently, the educated CAFs then secrete CXCL9/CXCL10 to stimulate CXCR3 on breast cancer cells, thereby maintaining the cancer stemness of breast cancer cells to promote lung metastatic colonization [11]. Interestingly, recent discoveries have also indicated that the tumor microenvironment in gallbladder cancer also contains an abundance of CAFs [12], however, the precise molecular function of CAFs in GBC and their effects on stemness maintenance and metastasis is poorly understood.

Thrombospondins (THBSs or TSPs) comprise a family of five adhesive glycoproteins that have prominent roles in cell-to-matrix and cell-to-cell interactions [13]. The family of thrombospondins can be divided into two subgroups: subgroup A TSPs (TSP-1 and TSP-2), which form trimers, and subgroup B TSPs (TSP-3, TSP-4 and TSP-5), which form pentamers. Previous studies have substantiated that different roles of TSPs in a variety of cancers [14], among these five thrombospondins, TSP-4 and TSP-5 are identified as the stromal glycoproteins highly expressed in CAFs of various types of cancers [13, 15]. Using laser-capture microdissection of reactive stromal tissues of prostate cancer for microarray expression analysis, the matrix interacting proteins TSP-4 and TSP-5 were shown to be notably upregulated in the reactive stroma of prostate cancer [15]. Similarly, via genome-wide transcriptome expression profiling analysis, TSP-4 was identified as being prominently upregulated in CAFs of diffuse-type gastric adenocarcinoma, which constitutes an abundance of the tumor stroma [13]. An emerging body of evidence has also demonstrated that hepatic stellate cell (HSC)-secreted TSP-5, which is also known as cartilage oligomeric matrix protein (COMP), stimulates CD36-mediated Akt/Erk signaling to promote liver fibrosis [16]. Furthermore, CAF-derived TSP-5 induces the epithelial-mesenchymal transition (EMT) and cancer stemness features of hepatocellular carcinoma [17]. Nevertheless, the regulatory mechanisms associated with the aberrant expression of thrombospondins in GBC stroma and their potential effects on GBC cells remain poorly understood and urgently needs to be elucidated.

In the present study, we aimed to elucidate the mechanisms associated with the tumor-stroma interaction-mediated proliferation, EMT and cancer stemness of cancer cells in GBC. The primary PTFs and CAFs from GBC tissues were isolated and used to screen the expression of thrombospondins, resulting in the identification of TSP-4 as a novel stromal molecule that was notably upregulated in GBC CAFs. CAF-derived TSP-4 was shown to interact with the transmembrane receptor integrin α2 on GBC cells, activating Akt-mediated heat shock factor1 (HSF1) signaling to promote the proliferation and induce the EMT and cancer stem-like traits of gallbladder cancer cells. Activated HSF1 signaling further increased the expression and paracrine of TGF-β1 to induce the transdifferentiation of PTFs into CAFs, recruit them into GBC, and foster TSP-4 expression as well. We found there existed a positive feedback loop between GBC cells and CAFs, and targeting this reciprocal interaction loop may be a promising strategy for conquering gallbladder cancer.

## Materials and methods

### Reagents

Recombinant human TSP-4 protein (rh-TSP-4), recombinant human TGF-β1 protein and a TGF-β-neutralizing antibody were obtained from R&D Systems (Minneapolis, MN, USA). An integrin α2 neutralizing antibody was purchased from Millipore (Darmstadt, Germany). LY294002 was obtained from Selleckchem (Houston, TX, USA). Detailed information for the antibodies used in the present study is displayed in Additional file 1: Table S1. All reagents were stored following the manufacturer’s instructions.

### Human GBC samples, CAFs isolation and cell cultures

Seventy-five gallbladder cancer tissues and adjacent nontumor tissues with corresponding clinical information of patients were obtained from the First Affiliated Hospital of Xi’an Jiaotong University. All samples were histopathologically confirmed and their corresponding patients had not received any chemotherapy before surgery. All patients provided written informed consent for the use of GBC samples. CAFs and peritumoral fibroblasts (PTFs) were isolated from the GBC tissues and adjacent nontumor gallbladder specimens of 15 patients, respectively, and cultured in F12/DMEM as previously described [12, 17]. All isolated CAFs and PTFs were used within 5 passages for experiments. The GBC cell lines, GBC-SD, SGC-996 and the normal biliary epithelial cell line, HIBEC, were purchased from the Shanghai Institute for Biological Science, Chinese Academy of Science (Shanghai, China). NOZ cells were obtained from the Health Science Research Resources Bank (Osaka, Japan). All cell lines were maintained in RPMI-1640 medium supplemented with 10% dialyzed fetal bovine serum (FBS) (HyClone, Logan, UT, USA), 100 μg/mL streptomycin and 100 U/mL penicillin.

### GBC-CAFs coculture model

A GBC-CAFs coculture model was established to evaluate the interplay between GBC cells and CAFs. The conditioned medium (CM) of GBC cells with or without HSF1 overexpression and that of CAFs with or without TSP-4 depletion was collected and filtered before being stored at − 80 °C for further use. To evaluate CAF-induced EMT and cancer stemness, serum-starved GBC cells were treated with the CM of CAFs. To investigate activated HSF1 signaling in GBC cells for the recruitment of fibroblasts, serum-starved PTFs were treated with the CM of GBC cells.

### Cell viability assay

After treatment with CM of CAFs with or without depletion of TSP-4 or rh-TSP-4, GBC cells were seeded into 96-well plates at a density of 5 × 10^3^ cells per well. Then, cell viability was assessed by the MTT assay at different time intervals (24, 48 and 72 h), with the absorbance measured at 490 nm with a multiwell microplate reader (BIO-TEC Inc., VA).

### Ethynyl deoxyuridine (EdU) incorporation assay

After co-cultured with CM of CAFs with or without TSP-4 depletion or rh-TSP-4, GBC cells were fixed in 4% formaldehyde for 30 min to undergo EdU incorporation assays using an EdU kit (Roche, Indianapolis, IN, USA) based on the manufacturer’s instructions. The samples were visualized with a Zeiss confocal microscope at a magnification of 200×, and images were captured in at least five random fields for further analysis.

### Colony formation assay

After plating the GBC cells at 1000 cells/well into 35-mm petri dishes and allowing them to attach overnight, the GBC cells were treated with for 24 h with the CM of CAFs with or without TSP-4 depletion or rh-TSP-4 treatment, after which the culture medium was removed and fresh medium was added. After 2 weeks of cultivation, cell colonies were fixed with 4% paraformaldehyde, stained with a 0.1% crystal violet solution, rinsed and then imaged.

### Enzyme-linked immunosorbent assay (ELISA) assay

After completing the designated interventions, the cells (CAFs or GBC cells) were serum-starved (1% serum in fresh medium) for an additional 48 h, after which the CM was collected and centrifuged (1500 rpm for 5 min). The secretion of TSP-4 and TGF-β into CM was detected using ELISA kits according to the manufacturer’s instructions (TSP-4 ELISA kit from Boster, Wuhan, China; TGF-β ELISA kit from R&D Systems, USA).

### Transwell migration and invasion assays

Migration and invasion assays were conducted as previously described [17, 18]. The CM collected from the supernatants of CAFs and recombinant human TSP-4 were used to treat GBC-SD and NOZ cells for invasion assays, while the CM collected from the supernatants of GBC-SD cells with or without HSF1 overexpression and recombinant human TGF-β were utilized to treat CAFs for migration assays.

### Tumorsphere formation assay

After being treated with the CM collected from CAFs or treatment with rh-TSP-4, GBC cells were plated at a density of 5000 cells per well in six-well ultralow attachment plates (Corning, Corning, NY, USA) and then cultured in serum-free DMEM/F12 medium (Gibco) containing 20 ng/mL of human FGF, 1% B27 (Invitrogen, Carlsbad, CA, USA), and 20 ng/mL of human EGF. The cells were subsequently incubated at 37 °C under an atmosphere with 5% CO_2_ for 14 days, after which the number and sizes of tumorspheres were counted or measured for tumorsphere formation analysis.

### RT-qPCR

Total RNA from CAFs was extracted using TRIzol reagents (Takara Bio, Dalian, China), and 1 μg RNA sample was reverse-transcribed into cDNA using a PrimeScript™ RT Reagent kit (Takara Bio). Quantitative reverse transcription PCR (RT-qPCR) was performed to assess the mRNA expression of target genes using the specific primers presented in Additional file 2: Table S2. GAPDH was used as a loading control, and the expression levels of genes were calculated using the 2^-ΔΔCt^ method.

### Western blot and coimmunoprecipitation (co-IP) assays

After the designated intervention, total proteins were extracted from whole-cell lysates with RIPA lysis buffer (Beyotime, Guangzhou, China), and the protein concentration of the samples was determined using a BCA protein assay kit (Pierce, Rockford, USA). The proteins in conditioned medium were extracted as previously described [19]. Western blot assays and densitometric analysis of the resulting protein bands were performed as previously described [19, 20]. For co-IP assays, GBC cells were lysed with lysis buffer, and the protein concentration of the cell lysates was determined by BCA quantitation. For each group, equal amounts of protein samples were incubated with protein G agarose beads (Catalogue number:17–0618-01 from GE Healthcare) and primary antibody or control immunoglobulin (IgG) overnight. Then, after five washes with lysis buffer, the precipitates were assessed by western blot analysis.

### Fluorescence-activated cell sorting (FACS)-ALDH activity

After co-culturing with the CM collected from CAFs or treatment with rh-TSP-4, an Aldefluor kit (Stem Cell Technologies, Vancouver, BC, Canada) was used to detect the ALDH enzymatic activity of GBC cells. NOZ or GBC-SD cells were suspended in Aldefluor assay buffer containing ALDH1 substrate and incubated for 30 min at 37 °C. The ALDH inhibitor diethylaminobenzaldehyde was used as negative control. The proportion of ALDH^+^ cells was examined by flow cytometry (BD FACS Canto II) and analyzed using FlowJo.

### Immunohistochemical (IHC) staining

The immunohistochemical (IHC) staining procedure was performed as previously described [21]. The percentages of positive stromal cells expressing TSP-4 were categorized as follows: 0 = < 10%, 1 = 10–25%, 2 = 25–50%, 3 = 50–75%, and 4 = > 75%. The staining intensity was scored as follows: 0 = no staining, 1 = light brown, 2 = brown, and 3 = dark brown. The final IHC score was determined by multiplying the staining intensity and the percentage of specifically positive staining tumor cells.

### Lentivirus and siRNA transfection

The lentiviral shRNA expression vector for TSP-4 or SMAD3 and the control pLKO.1 plasmid were purchased from Addgene (Cambridge, MA, USA). pcDNA3.1-Control (Vector) and pcDNA3.1-HSF1 (OE-HSF1) were purchased from Shanghai GenePharma Co., Ltd. (Shanghai, China). The lentiviruses were generated as previously described [22]. Virus-containing medium from HEK293T cells (Invitrogen) was collected 48 and 72 h after transfection. CAFs or GBC cells were infected by incubation with the lentivirus-containing medium and were subsequently treated with puromycin to select for knockdown cells. The specific small interfering RNA (siRNA) against HSF1 (HSF1-Homo-380: sense GCGGCAGCUCAACAUGUAUTT, antisense AUACAUGUUGAGCUGCCGCTT), and a negative control siRNA (sense UUCUCCGAACGUGUCACGUTT, antisense ACGUGACACGUUCGGAGAATT) were obtained from GenePharm (Shanghai, China). The siRNAs were transfected into GBC cells using lipofectamine RNAi MAX (Invitrogen) following the manufacturer’s instructions.

### In vivo tumorigenesis assays

All animal experiments were conducted according to the protocols authorized by the Ethics Committee of Xi’an Jiaotong University. To establish the CAF and GBC cell coinjection model, sh-vector or sh-TSP-4 lentiviruses were first transfected into CAFs. Then, single-cell suspensions of 5 × 10^5^ of GBC-SD cells alone or together with CAFs (sh-vector or sh-TSP-4) were mixed at a ratio of 1:1 in 100 mL of PBS, after which they were subcutaneously injected into the left flanks of 4-week-old male BALB/c nude mice (acquired from and housed in the Animal Center at Medical College, Xi’an Jiaotong University). Tumor growth was continuously monitored by calculating the tumor volume according to the following formula: V (tumor volume) =0.5 × s (shorter diameter)^2^ × L (longer diameter). The mice were sacrificed at day 28, and the tumor specimens were weighed, measured and underwent immunohistochemical (IHC) staining for histological analyses. To determine whether HSF1-expressing GBC cells can recruit CAFs into GBC tumors, GBC-SD cells were transfected with vector or OE-HSF1 lentiviruses and then subcutaneously injected into the left flanks of 4-week-old male BALB/c nude mice. Four weeks later, the mice were sacrificed and the tumor samples were analyzed by IHC staining to assess α-SMA expression.

### Statistical analysis

The data are displayed as the means±SD of three independent experiments. SPSS 18.0 was used to analyze data with a normal distribution and equal variance. Comparisons between two groups were conducted using Student’s t-test, and differences among multiple groups were assessed by one-way ANOVA followed by the LSD post hoc test. The survival of different groups was evaluated by Kaplan-Meier analysis. *P* < 0.05 was regarded as a statistically significant difference.

## Results

### TSP-4 is predominantly expressed in CAFs of GBC and predicts poor prognosis

**Fig. 1:**
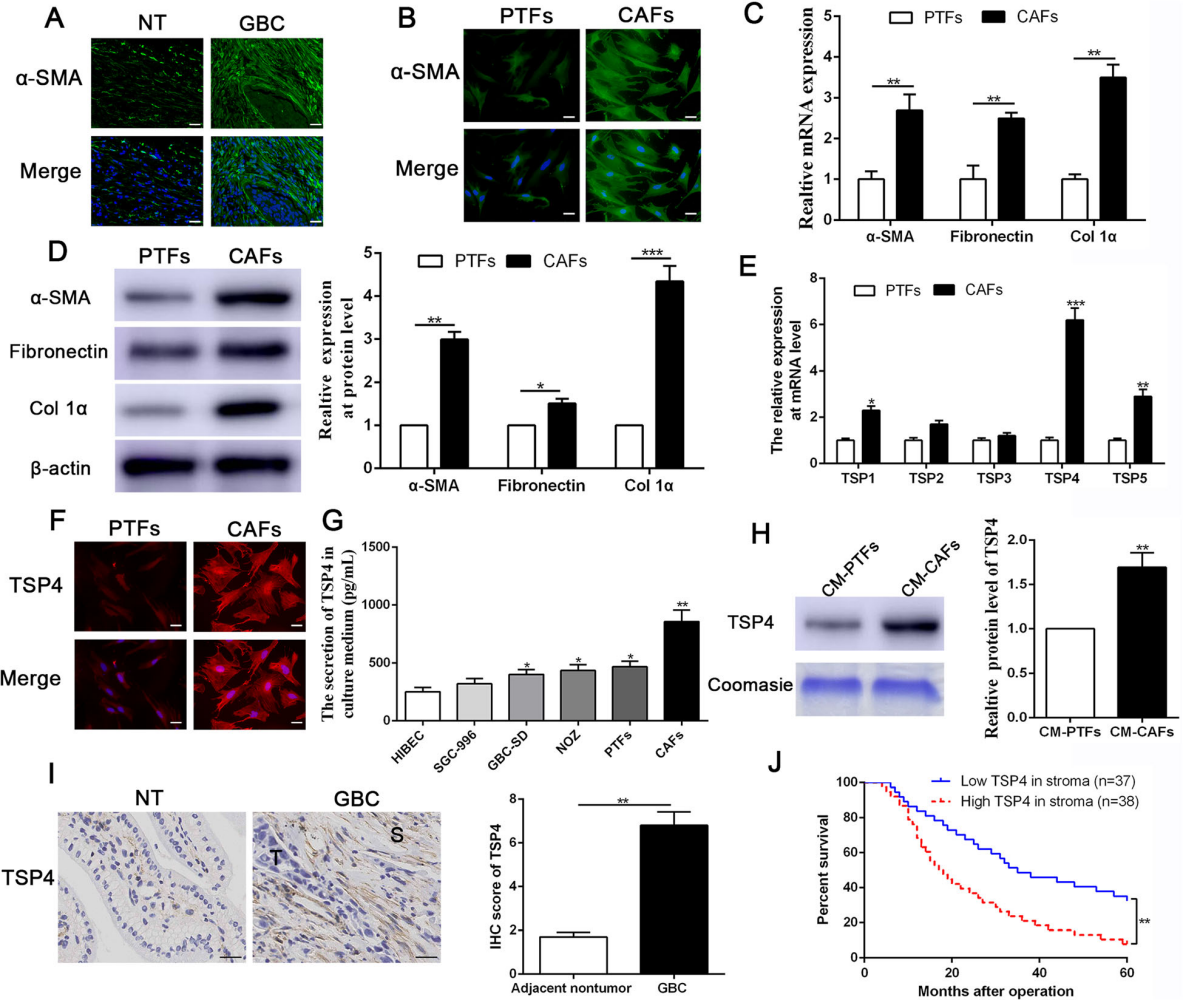
TSP-4 is mainly derived from CAFs in GBC and predicts poor prognosis. **a** Immunofluorescence (IF) staining of α-SMA in human GBC and adjacent non-tumor (NT) tissue. The magnification of the picture is 400×. Scale bars = 20 μm. **b** CAFs and PTFs were isolated from human GBC tissues and adjacent non-tumor tissue. α-SMA level in CAFs and PTFs were assessed by IF staining. The magnification of the picture is 400×. Scale bars = 20 μm. **c**, **d** The expression of α-SMA, fibronectin and col. 1α at mRNA and protein level was determined by qRT-PCR and Western blot respectively. *n* = 15, *P < 0.05 or **P < 0.01 by Student’s t-test. **e** qRT-PCR was used to screen the expression of thrombospondins (TSP1-TSP5) in CAFs and PTFs. *n* = 15, *P < 0.05 or **P < 0.01 or ***P < 0.001 by Student’s t-test. **f** TSP-4 level in CAFs and PTFs were determined by IF staining. The magnification is 400×. Scale bars = 20 μm. **g** The secretion of TSP-4 in GBC cell lines, PTFs and CAFs was confirmed by Elisa assay. n = three independent experiments, *P < 0.05 or **P < 0.01 by ANOVA. **h** The secretion of TSP-4 in CM-PTFs and CM-CAFs was assessed by western blot. *n* = 15, *P < 0.05 or **P < 0.01 by Student’s t-test. **i** The expression of TSP-4 was upregulated in stroma of GBC compared to that in normal tissues, as determined by IHC staining. **P < 0.01 by Student’s t-test. **j** Kaplan-Meier survival curves of overall survival in our GBC patients’ cohort (*n* = 75). Patients were assigned into two subgroups according to the median expression of TSP-4 in GBC stroma. **P < 0.01by two-sided log-rank test

To confirm the presence of CAFs in gallbladder cancer tissues, immunofluorescence staining was first performed to visualize α-SMA expression in GBC samples. As shown in Fig. 1a, more α-SMA + -stained cells were observed in GBC tissues than in adjacent nontumor tissues, indicating the presence of abundant CAFs in GBC. Then, we isolated CAFs and PTFs from GBC specimens and adjacent nontumor tissues respectively, and the identification of CAFs was verified by IF staining, RT-qPCR and western blot analyses. As shown in Fig. 1b-d, higher expression levels of CAF markers (α-SMA, fibronectin and collagen Iα) were observed in CAFs than PTFs, consistent with the results of previous studies [12]. To directly explore the expression mode of thrombospondins (TSPs) in the reactive stroma of GBC, we utilized RT-qPCR to screen a panel of TSPs in CAFs and identified TSP-4 mRNA as the most predominantly upregulated TSP in CAFs derived from GBC patients when compared with PTFs (Fig. 1e). IF staining results also confirmed high expression level of TSP-4 in CAFs (Fig. 1f). We next performed ELISA to assess the secretion of TSP-4 in GBC cell lines, PTFs and CAFs. As shown in Fig. 1g, the TSP-4 concentration was significantly higher in the supernatants of CAFs than in those of GBC cells or PTFs. Similarly, western blot results also indicated that the secretion of TSP-4 was notably enhanced in the CM of CAFs compared to that of PTFs (Fig. 1h). Immunohistochemical analysis of TSP-4 expression in gallbladder cancer and adjacent nontumor tissues revealed remarkably positive staining in the reactive stroma of GBC (Fig. 1i). To further validate the clinical importance of TSP-4 in GBC, we performed Kaplan-Meier analysis of gallbladder cancer patients and observed that high TSP-4 expression in GBC stroma predicted poor prognosis with shorter overall survival in 75 GBC patients (Fig. 1j). Furthermore, as shown in Additional file 3: Table S3, high TSP-4 expression in stroma was detected at a significantly greater frequency in tissues with increased tumor sizes (*P* = 0.006) and lymph node metastasis (*P* = 0.001).

### CAF-derived TSP-4 promotes GBC cell proliferation

To directly assess whether CAF-derived TSP-4 impacted the malignant phenotypes of GBC cells, NOZ and GBC-SD cells were used for the following experiments. TSP-4 depletion in CAFs was performed using TSP-4^shRNA^ lentiviruses, and the depletion efficiency was evaluated by western blot analysis. As shown in Additional file 5: Fig. S2A, TSP-4 protein expression was lower in TSP-4^shRNA^ cells than in shVector control cells, and we selected the TSP-4 ^shRNA#2^ for further experiments. CAFs conditioned medium (CM_CAFs_) intervention significantly enhanced the proliferation capacity of NOZ and GBC-SD cells, as confirmed by MTT, colony formation and Edu assays (Additional file 4: Fig. S1A-1D). Then, the conditioned medium obtained from CAFs with TSP-4 depletion (CM_CAFs-shTSP-4_) was used to treat NOZ and GBC-SD cells, and the results indicated that TSP-4 knockdown hindered the promotion-effects of CAFs on GBC cells proliferation (Additional file 4: Fig. S1A-1D). To further investigate whether paracrine TSP-4 signaling has a role in the mechanism by which CAFs induce GBC cell proliferation, exogenous rh-TSP-4 were utilized to regulate the concentration of TSP-4 in the CM of CAFs. Interestingly, administration of exogenous rh-TSP-4 (25 nM) into CM_CAFs-shTSP-4_ rescued the TSP-4 depletion-induced decrease in GBC cell proliferation (Additional file 4: Fig. S1A-1D). Additionally, treatment of GBC cells with rh-TSP-4 alone could also promote the proliferation of NOZ and GBC-SD cells (Additional file 5: Fig. S2B-S2D). Collectively, these data convincingly demonstrate that CAFs derived TSP-4 promotes GBC cell proliferation.

### Paracrine TSP-4 produced by CAFs enhances the EMT and cancer stem-like properties of GBC cells

**Fig. 2:**
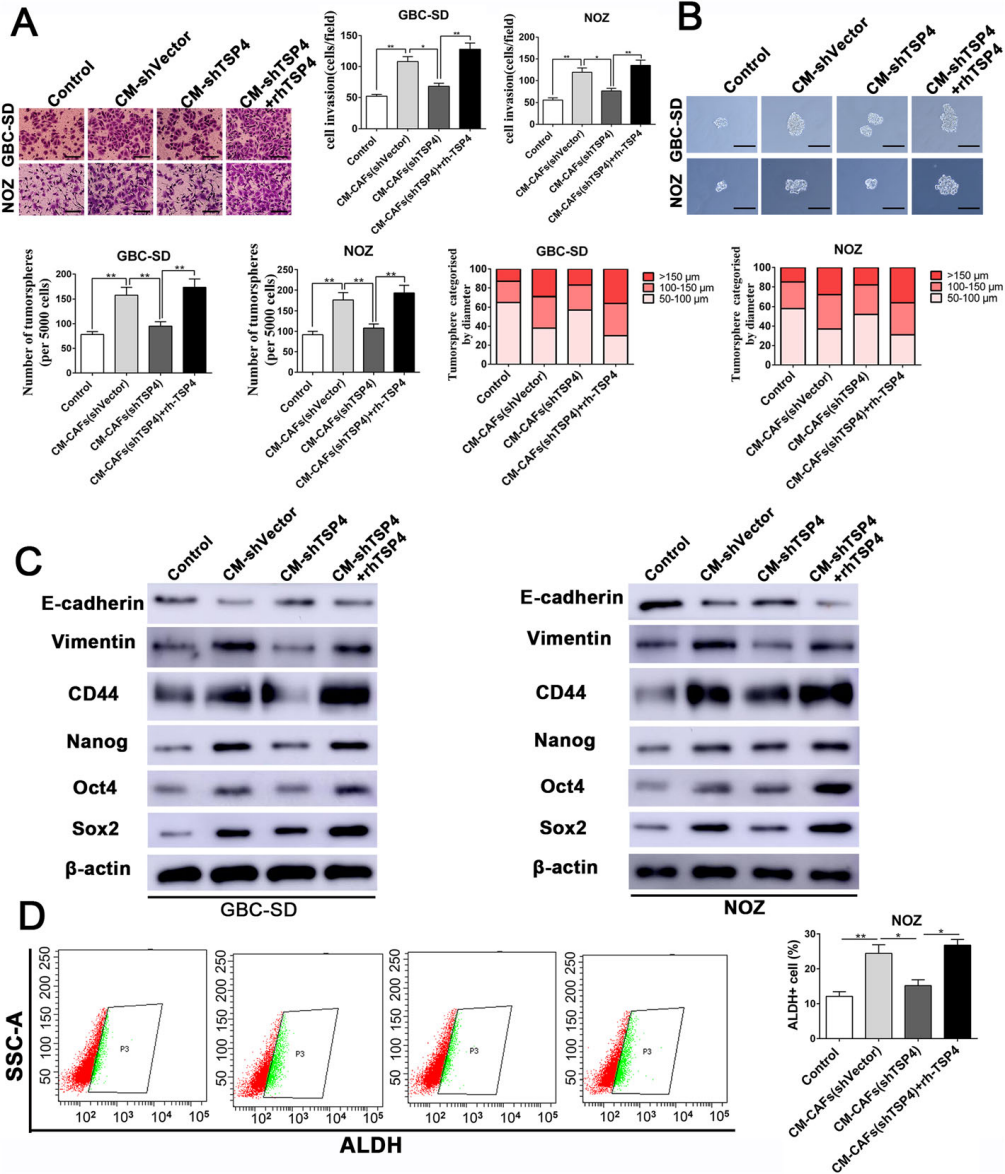
CAFs-derived TSP-4 signaling facilitated EMT and cancer stem-like traits in GBC cells. **a** GBC-SD and NOZ cells were incubated with CM-shVector, CM-shTSP-4, CM-shTSP-4 + rh-TSP-4 for 24 h, then the invasive ability of GBC cells was assessed by the Matrigel-invasion assay. Scale bars = 50 μm. n = three independent experiments, *P < 0.05 or **P < 0.01 by ANOVA. **b** Representative images of the tumorsphere formation assay after CM-shVector, CM-shTSP-4, CM-shTSP-4 + rh-TSP-4 treatments in GBC-SD and NOZ cells. The number of tumorspheres was counted and plotted, and the percentage of tumorspheres with diameters of 50–100 μm, 100–150 μm or > 150 μm was calculated and plotted. Magnification is × 200, and scale bars = 50 μm. n = three independent experiments, **P < 0.01 by ANOVA. **c** The expression of CSC and EMT markers after CM-shVector, CM-shTSP-4, CM-shTSP-4 + rh-TSP-4 treatments were evaluated by western blotting. **d** The ALDH+ cells populations in NOZ cells after CM-shVector, CM-shTSP-4, CM-shTSP-4 + rh-TSP-4 treatments were detected by Flow cytometric analysis. n = three independent experiments, *P < 0.05 or **P < 0.01 by ANOVA

Owing to the evidence supporting the impact of CAFs on the promotion of invasive potential and cancer stem-like traits, we postulated that the paracrine of TSP-4 by CAFs would promote these properties. We tested this hypothesis by depleting TSP-4 in CAFs and then collecting their CM to establish an indirect coculture system. The functional assay results showed that CAFs CM could significantly enhance the invasion and tumorsphere-formation capacities of GBC cells, while GBC cells treated with CM from TSP-4 knockdown CAFs exhibited reduced invasive potential and tumorsphere-formation capacity (Fig. 2a-b). Moreover, the CM_CAFs_ treatment induced the expression of EMT and cancer stemness markers in NOZ and GBC-SD cells, as revealed by reduced E-cadherin expression and increased vimentin, Sox2, CD44, Nanog and Oct4 expression (Fig. 2c and Additional file 4: Fig. S1F). Analogously, these effects of CM_CAFs_ were also partly impeded by TSP-4 knockdown (Fig. 2c and Additional file 4: Fig. S1F). In addition, the CM_CAFs_ co-cultured with GBC cells induced an increase of the aldehyde dehydrogenase (ALDH) activity, an important marker of cancer stemness, in NOZ and GBC-SD cells, while ALDH activity was partly inhibited upon in GBC cells treated with CM_CAFs-shTSP-4_ (Fig. 2d and Additional file 4: Fig. S1E). The supplementation of CM_CAFs-shTSP-4_ with rh-TSP-4 could reverse the effects of TSP-4 depletion in CAFs induced the decreased the EMT and cancer stem-like characteristics of GBC cells, as determined by invasion, tumorsphere-formation, western blot and ALDH activity assays (Fig. 2a-d and Additional file 4: Fig. S1E-S1F). Moreover, treatment with NOZ and GBC-SD cells with rh-TSP-4 alone also facilitated their invasion and cancer stem-like properties (Additional file 5: Fig. S2E-S2G), and the presence of rh-TSP-4 prominently promoted the expression of EMT and cancer stemness markers in NOZ and GBC-SD cells in a dose-dependent manner, as confirmed by western blot analysis (Additional file 5: Fig. S2H).

**Fig. 3:**
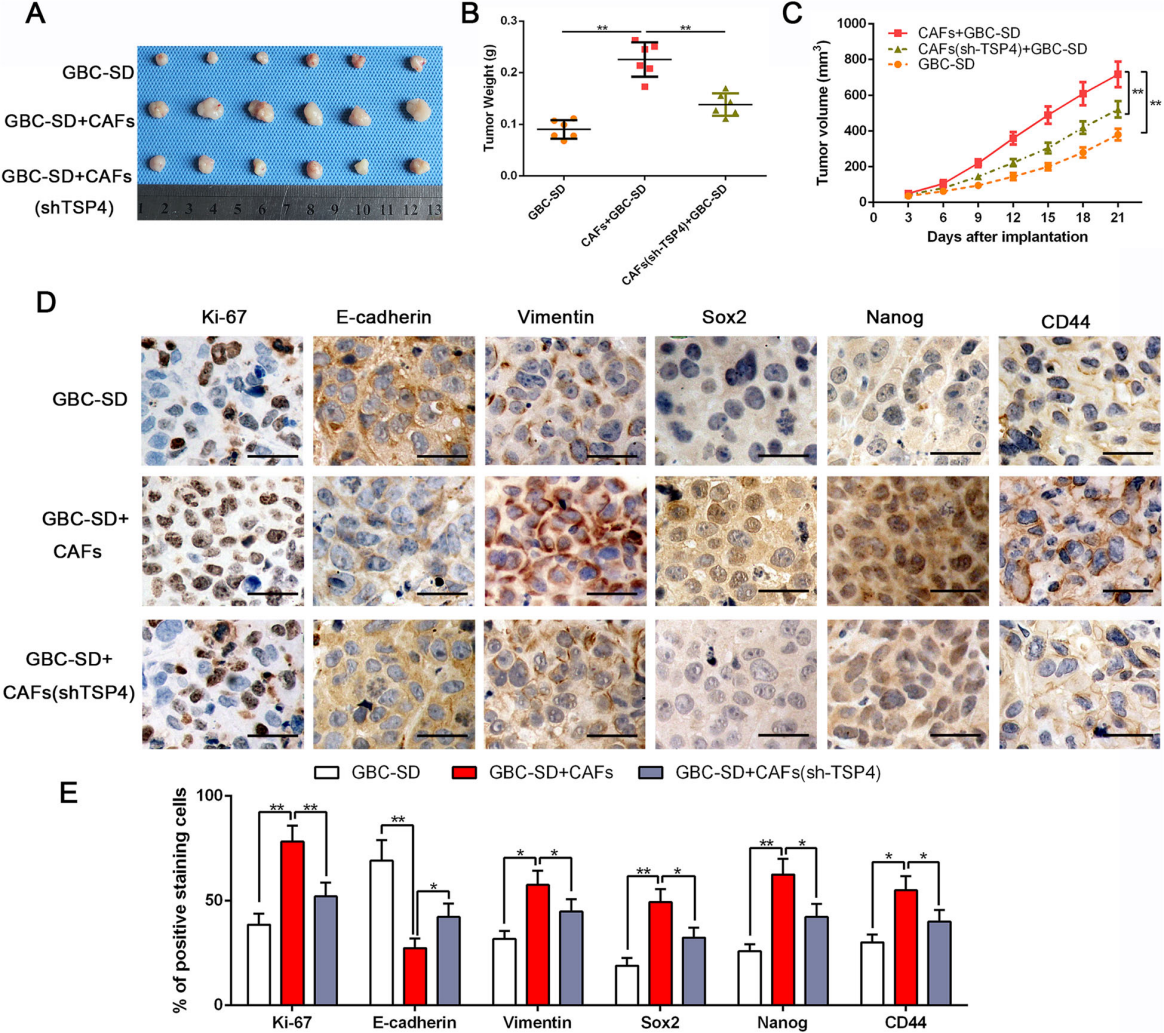
Depletion of TSP-4 in CAFs dampens CAFs-induced tumorgenicity of GBC-SD cells along with reversing the mesenchymal and stem-like phenotypes. **a** Representative images of subcutaneous xenografts in nude mice implanted with GBC-SD alone, GBC-SD + CAFs and GBC-SD + CAFs (shTSP-4) (*n* = 6). **b**, **c** Xenografts weight (mg) and tumor sizes were monitored and undergone quantification analysis. *n* = 6, **P < 0.01 by ANOVA for tumor weight; **P < 0.01 by repeated-measures ANOVA for tumor sizes. **d**, **e** Immunohistochemistry staining and semiquantitative analysis of Ki-67, E-cadherin, Vimentin, Sox2, Nanog and CD44 in xenograft tissues from different groups. Magnification is × 400, the scale bar represents 20 μm. *n* = 6, *P < 0.05 or **P < 0.01 by ANOVA

CSCs feature stem cell-like properties, such as self-renewal, tumor initiation, growth, invasion and metastasis [23]. Therefore, we explored whether CAFs could enhance in vivo tumorigenesis by subcutaneously injecting nude mice with GBC-SD cells and CAFs at a 1:1 ratio. Mice coimplanted with GBC-SD cells and CAFs generated tumors with higher weights and volumes than those injected with GBC-SD cells alone (*P* < 0.05, Fig. 3a-c). Similarly, the coinjection of TSP-4-depleted CAFs with GBC-SD cells clearly blunted the tumorigenesis-promoting effect of CAFs (*P* < 0.05, Fig. 3a-c). IHC staining of tumors from mice coinjected with CAFs/GBC-SD and a semiquantitative analysis revealed a strikingly higher expression of Ki-67, EMT and cancer stemness markers in tumor cells than was observed in subcutaneous tumors from mice injected with GBC-SD cells alone (Fig. 3d-e). In contrast, TSP-4 knockdown in CAFs partly reduced the expression levels of Ki-67, EMT and CSC makers in tumors from mice coinjected with TSP-4-depleted CAFs/GBC-SD (Fig. 3d-e). Collectively, our in vivo data provided support to our in vitro findings and confirmed that important roles of TSP-4 in mediating tumor-stroma interactions to modulate the proliferation, EMT and cancer stem-like characteristics of gallbladder cancer.

### Integrin α2 mediates the effects of paracrine TSP-4 signaling

**Fig. 4:**
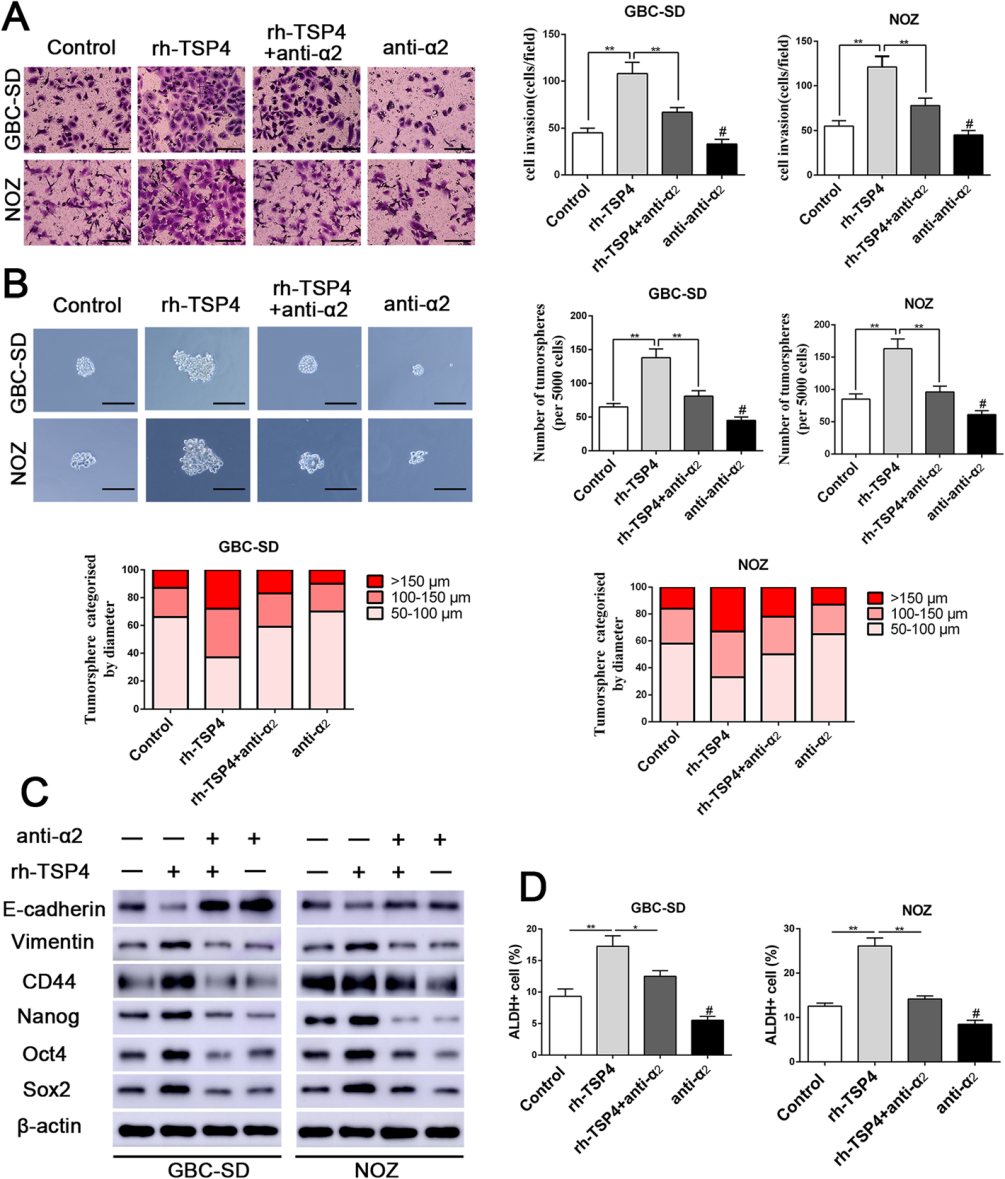
Integrin α2 mediates the efficacy of paracrine of TSP-4 signaling on EMT and cancer stemness of GBC cells. **a** Representative images of the Matrigel invasion assay after rh-TSP-4, rh-TSP-4 + anti-α2 or anti-α2 treatments in GBC-SD and NOZ cells. Scale bars = 50 μm. n = three independent experiments, **P < 0.01 or ^#^ *P* < 0.01by ANOVA versus control group. **b** Representative images of the tumorsphere formation assay after rh-TSP-4, rh-TSP-4 + anti-α2 or anti-α2 treatments in GBC-SD and NOZ cells. The number of tumorspheres was counted and plotted, and the percentage of tumorspheres with diameters of 50–100 μm, 100–150 μm or > 150 μm was calculated and plotted. Magnification is × 200, and scale bars = 50 μm. n = three independent experiments, **P < 0.01 or ^#^ *P* < 0.01by ANOVA versus control group. **c** The expression of CSC and EMT markers after rh-TSP-4, rh-TSP-4 + anti-α2 or anti-α2 treatments were evaluated by western blotting. **d** The ALDH+ cells populations after rh-TSP-4, rh-TSP-4 + anti-α2 or anti-α2 treatments were detected by Flow cytometric analysis. n = three independent experiments, *P < 0.05 or **P < 0.01 or ^#^ *P* < 0.01by ANOVA versus control group

Previous studies have substantiated that integrin α2 as a receptor of TSP-4 exerts a crucial role in TSP-4-mediated malignant tumor progression [24]. To further elucidate the molecular mechanisms by which integrin α2 receptor participates in paracrine TSP-4 signaling, a specific integrin α2-neutralizing antibody was used in subsequent experiments. The specific integrin α2-neutralizing antibody was able to mitigate the rh-TSP-4-induced proliferation, invasion, and tumorsphere-forming capacities of GBC cells (Additional file 6: Fig. S3A-S3C and Fig. 4a-b). Similarly, the integrin α2-neutralizing antibody also attenuated the rh-TSP-4-induced expression of EMT and cancer stemness markers in GBC cells, as determined by western blot analysis (Fig. 4c and Additional file 6: Fig.S3D). Additionally, ALDH activity analysis by flow cytometry also corroborated that integrin α2-neutralizing antibody was able to abrogate the rh-TSP-4-induced increase in ALDH activity (Fig. 4d). These results demonstrated the importance of integrin α2 in paracrine TSP-4-midiated facilitation of proliferation, EMT and cancer stemness.

### P-HSF1(S326) is induced by TSP-4 to drive GBC cell proliferation, EMT and cancer stemness

**Fig. 5:**
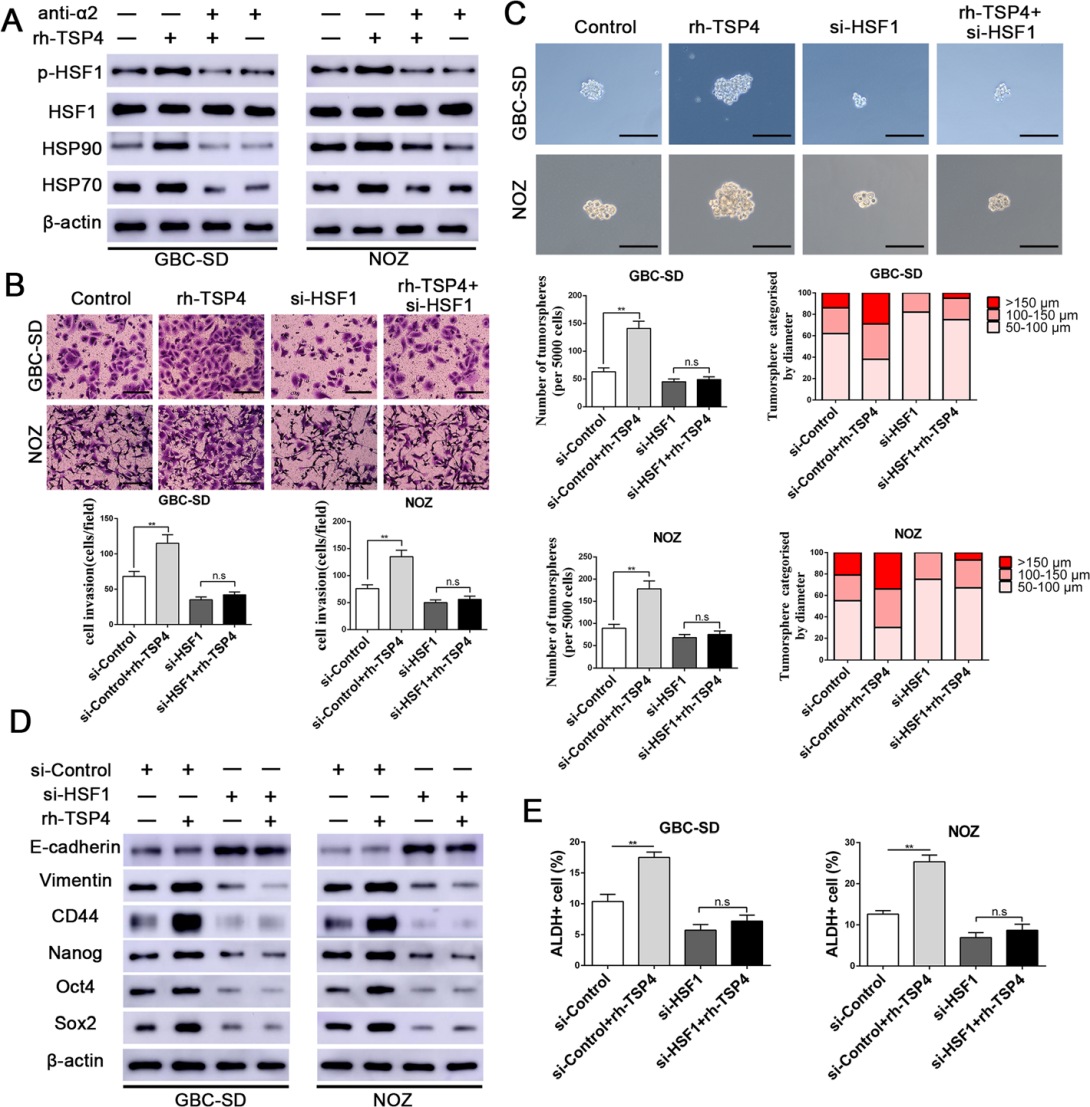
HSF1 activation mediates the effects of the TSP-4/integrin α2 axis on EMT and CSC-like features in GBC cells. **a** The protein expression levels of p-HSF1 (sc326), HSF1 and its downstream targets after rh-TSP-4, rh-TSP-4 + anti-α2 or anti-α2 treatments were determined by western blot analysis in GBC-SD and NOZ cells. **b** Representative images of the Matrigel invasion assay after rh-TSP-4, si-HSF1or rh-TSP-4 + si-HSF1 treatments in GBC-SD and NOZ cells. Scale bars = 50 μm. n = three independent experiments, **P < 0.01 by ANOVA versus si-control group. **c** Representative images of the tumorsphere formation assay after rh-TSP-4, si-HSF1or rh-TSP-4 + si-HSF1 treatments in GBC-SD and NOZ cells. The number of tumorspheres was counted and plotted, and the percentage of tumorspheres with diameters of 50–100 μm, 100–150 μm or > 150 μm was calculated and plotted. Magnification is × 200, and scale bars = 50 μm. n = three independent experiments, **P < 0.01by ANOVA versus si-control group. **d** The expression of EMT and CSC markers (E-cadherin, Vimentin, CD44, Nanog, Oct4 and Sox2) after rh-TSP-4, si-HSF1or rh-TSP-4 + si-HSF1 treatments were evaluated by western blotting. **e** The ALDH+ cells populations after rh-TSP-4, si-HSF1or rh-TSP-4 + si-HSF1 treatments were detected by Flow cytometric analysis. n = three independent experiments, **P < 0.01by ANOVA versus si-control group

HSF-1 phosphorylation at S326 has been demonstrated to activate HSF-1 transcriptional activity [25, 26]. To elucidate the downstream effector of the paracrine TSP-4/integrin α2 axis and investigate the potential efficacy of HSF1 in TSP-4-induced malignant phenotypes in GBC cells, we first evaluated p-HSF1(S326) and its downstream targets (HSP90 and HSP70) after treatment with rh-TSP-4 or integrin α2 blockage. As shown in Fig. 5a and Additional file 7: Fig.S4A, the levels of p-HSF1(S326) and its downstream targets (HSP90 and HSP70) were notably increased after rh-TSP-4 stimulation, while their expression was dampened by pretreatment with an integrin α2-neutralizing antibody. With the aim to further assess whether HSF1 is the downstream effector of the paracrine TSP-4/integrin α2 axis, NOZ and GBC-SD cells were transfected with si-HSF1 then incubated with rh-TSP-4 for 24 h. We observed that rh-TSP-4 treatment increased the proliferation, invasion, EMT as well as elevated cancer stem-like features of GBC-SD and NOZ cells, while the rh-TSP-4-mediated induction of these effects were abolished by HSF1 silencing (Fig. 5b-e and Additional file 7: Fig. S4B-S4E). Collectively, these results indicate that the proliferation, invasion and cancer stem-like characteristics promoted by CAF-derived TSP-4 is achieved by activation of HSF1 signaling.

### TSP-4/integrin α2 axis-induced phosphorylation of HSF1 at S326 is mediated by Akt activation in GBC cells

**Fig. 6:**
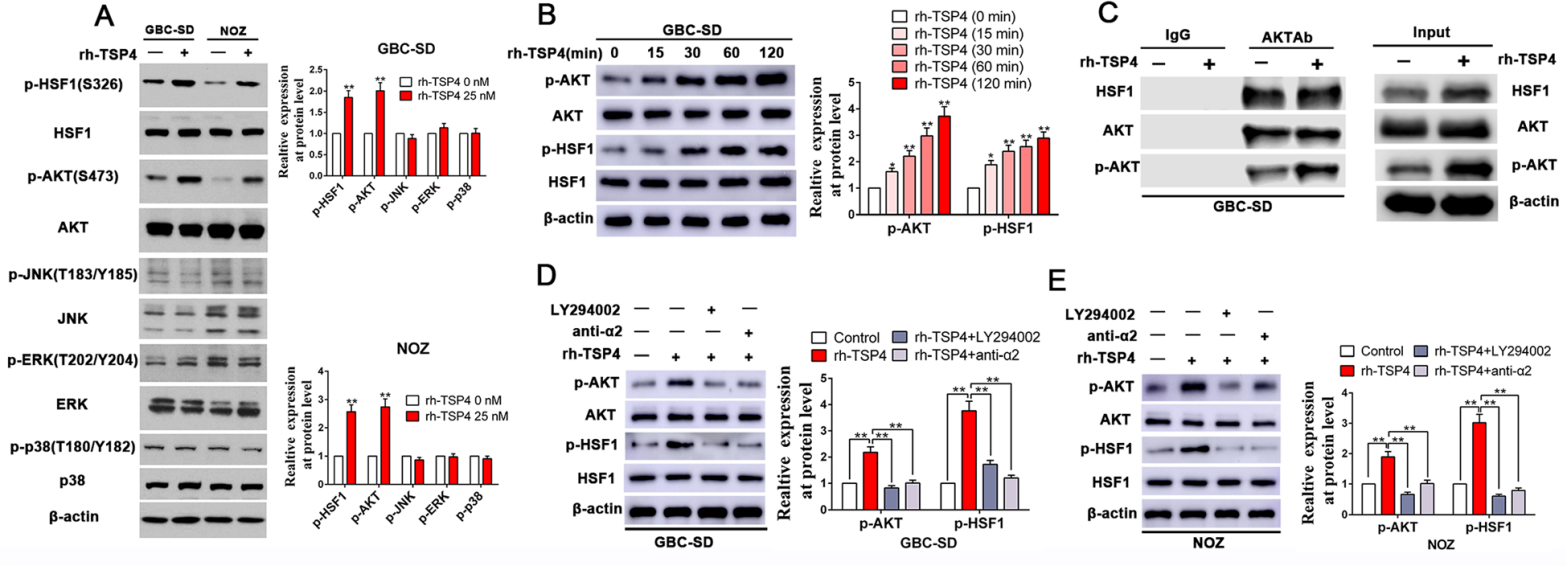
Concurrent activation of HSF1 and AKT by TSP-4/integrin α2 axis in gallbladder cancer cells. **a** rh-TSP-4 induced phosphorylation of both AKT and HSF1 ((p-AKT: S473 and p-HSF1:S326)) in GBC cells. GBC-SD and NOZ cells were treated with and without rh-TSP-4 for 2 h and the whole cell lysates were analyzed by WB for levels of integrin α2 downstream kinases. β-Actin was used as an internal control. n = three independent experiments, **P < 0.01 by Student’s t-test versus control group. **b** Kinetics for HSF-1 activation was in accordance with that for Akt. GBC-SD cells were incubated with rh-TSP-4 for 0–120 min and the whole cell lysates were utilized for WB analysis to determine levels of p-HSF-1 (S326) and p-Akt (S473). β-Actin was used as an internal control. n = three independent experiments, **P < 0.01 by ANOVA versus control group. **c** Akt interacts with HSF-1 constitutively, independent of rh-TSP-4 treatment. CO-IP assay was performed using whole cell lysates extracted from GBC-SD cells treated with and without rh-TSP-4. An Akt antibody was used to immunoprecipitate Akt, whereas IgG was used as negative controls. **d**, **e** Western blot analysis showed that rh-TSP-4 increased Akt and HSF1 phosphorylation (p-AKT: S473 and p-HSF1:S326) in GBC-SD and NOZ cells, while this effect was antagonized by blocking integrin α2 or inhibiting Akt. β-Actin was used as an internal control. n = three independent experiments, **P < 0.01 by ANOVA versus control group

The TSP-4/integrin α2 axis is well known to activate a number of downstream signaling molecules, such as PI3K/Akt, mTOR ERK, p38, and JNK. Therefore, we evaluated which of these downstream signaling molecules of the TSP-4/integrin α2 axis are activated in concordance with HSF-1 activation. As shown in Fig. 6a, simultaneous phosphorylation was observed for HSF-1 at S326 with Akt at S473 but not with the other assayed kinases [p-ERK (T202/Y204), p-JNK (T183/Y185), and p-mTOR (S2448, p-p38: T180/Y182)]. To further verify the results with simultaneous activation of HSF-1 and Akt, GBC-SD cells were treated with rh-TSP-4 for 0–120 min and assessed for the levels of p-HSF-1 (S326) and p-Akt (S473). The results indicated that the kinetics for HSF-1 activation is in accord with that for Akt, which were both in time-dependent manner for rh-TSP-4 induced phosphorylation (Fig. 6b). Next, we were aspired to investigate whether these two proteins physically associated. Through CO-IP assays, we confirmed that Akt directly interacts with HSF-1 independent of rh-TSP-4 treatment (Fig. 6c). Furthermore, we interrogated the role and requirement of Akt signaling in TSP-4-mediated HSF1 activation facilitating the proliferation, EMT and cancer stemness of GBC cells. As displayed in Fig. 6d-e, rh-TSP-4 treatment enhanced Akt phosphorylation (S473), but this effect was notably repressed by pretreatment with integrin α2-neutralizing antibody or the PI3K inhibitor LY294002. To assess the involvement of Akt activation in the paracrine TSP-4/integrin α2 axis-induced activation of HSF1, we further measured the changes in p-HSF1(S326) levels in response to rh-TSP-4 after pretreating NOZ and GBC-SD cells with the PI3K inhibitor LY294002. The results demonstrated that PI3K inhibition attenuated the paracrine TSP-4/integrin α2 axis-induced increase in p-HSF1(S326) levels in NOZ and GBC-SD cells (Fig. 6d-e). Unsurprisingly, the promotion effects on cell proliferation, EMT and cancer stemness mediated by rh-TSP-4 stimulation were partly counteracted by PI3K inhibition (Additional file 8: Fig. S5 and Additional file 9: Fig. S6).

### HSF1-mediated TGF-β signaling induces the transdifferentiation of PTFs into CAFs and promotes their recruitment to GBC

**Fig. 7:**
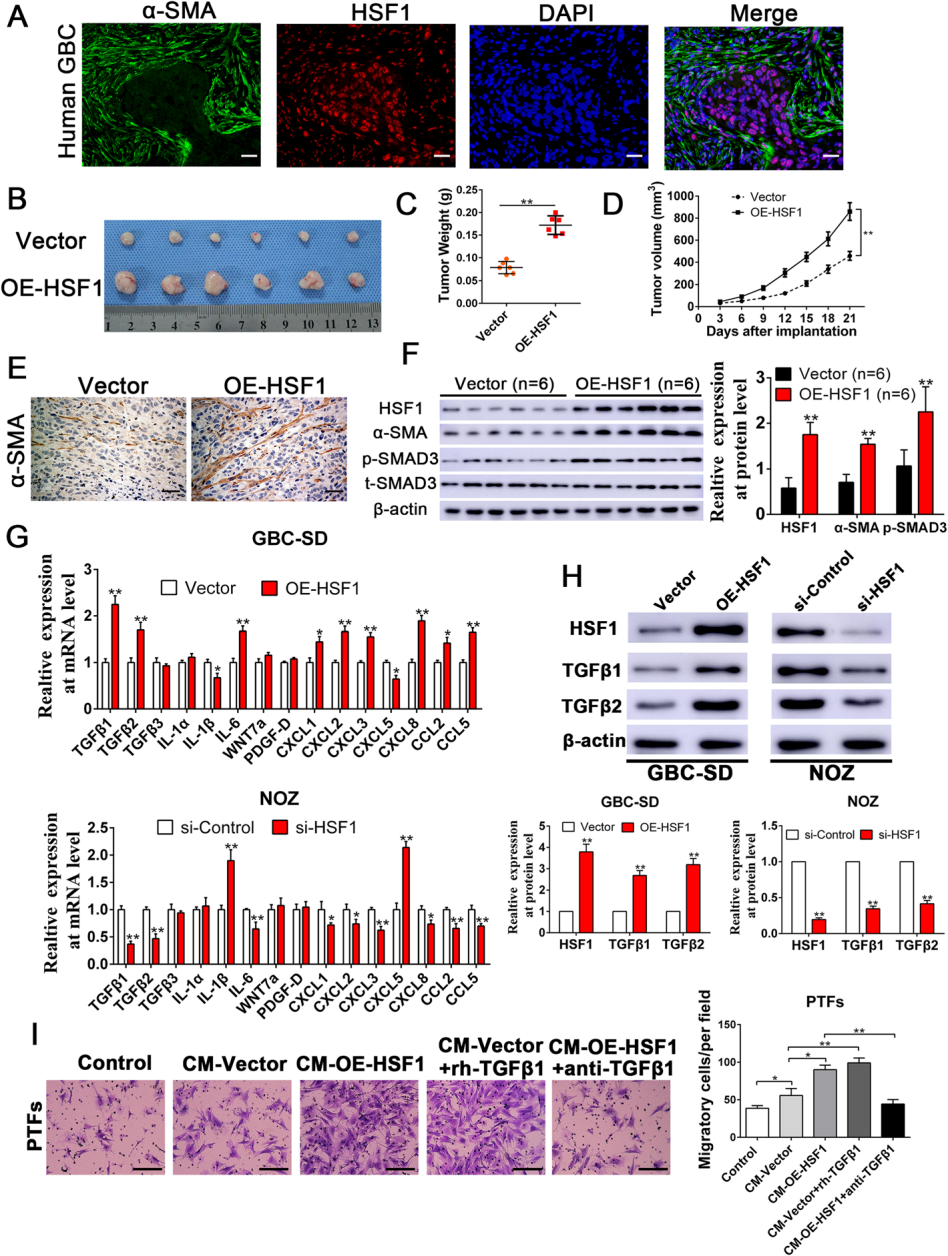
Overexpression of HSF1 in GBC induced the recruitment of CAFs through TGFβ signaling. **a** The double IF staining in human GBC tissue displayed that HSF1 was principally expressed in nuclear of gallbladder cancer cells, while α-SMA positive stroma was surrounded in HSF1 positive GBC cells. **b** Representative images of subcutaneous xenografts in nude mice implanted with GBC-SD cells with Vector or overexpression of HSF1 group (*n* = 6). **c**, **d** Xenografts weight (mg) and tumor sizes were monitored and undergone quantification analysis. *n* = 6, **P < 0.01 by Student’s t-test for tumor weight; **P < 0.01 by repeated-measures ANOVA for tumor sizes. **e** IHC analyses verified that α-SMA expression levels were significantly increased in xenograft tumors from nude mice subcutaneous implantation models of GBC-SD cells expressing exogenous HSF1. Magnification is × 400, the scale bar represents 20 μm. **f** Xenograft tissues arising from HSF1 overexpression group (*n* = 6) and control group (*n* = 6) were subjected to immunoblotting for HSF1, α-SMA, p-SMAD3 and t-SMAD3 protein expression, respectively. *n* = 6, **P < 0.01 by Student’s t-test. **g** The expression patterns in mRNA levels of the selected chemokines, inflammation-related genes and TGFβ after manipulation of HSF1 in GBC cells. n = three independent experiments, *P < 0.05 or **P < 0.01 by Student’s t-test. **h** The alterations of TGFβ1 and TGFβ2 expression levels were assessed by Western blot in GBC-SD and NOZ cells after manipulations of HSF1. β-Actin was used as an internal control. n = three independent experiments, **P < 0.01 by Student’s t-test. **i** The migration capacity of PTFs in response to CM-Vector, CM-OE-HSF1, CM-Vector+TGFβ1 or CM-OE-HSF1 + anti-TGFβ treatment was detected by Transwell-migration assay. Scale bars = 50 μm. n = three independent experiments, *P < 0.05 or **P < 0.01 by ANOVA

To determine whether activated HSF1 signaling may feedback to promote the recruitment of infiltrating CAFs, double IF staining was performed to detect α-SMA and HSF1 in human gallbladder cancer specimens. The results showed that tumor cells in GBC samples were surrounded by an abundance of CAFs staining positive for α-SMA, while HSF1 was mostly expressed in gallbladder cancer cells and primarily translocated into cell nuclei, indicating a reactive stroma in GBC and that activated HSF1 signaling may exert an important role in CAFs recruitment (Fig. 7a). To further examine the efficacy of HSF1 on CAFs recruitment in vivo, OE-HSF1 (overexpressing HSF1) GBC-SD and vector control cells were subcutaneously injected into nude mice. The weights and the volumes of the tumors from mice injected with OE-HSF1 GBC-SD cells were dramatically higher than those observed in the control group (Fig. 7b-d), indicating that HSF1 overexpression remarkably increased the tumorigenic ability of GBC cells in vivo. IHC staining was performed to examine whether there were increased numbers of CAFs expressing α-SMA in GBC-SD xenograft tumors with enforced HSF1 expression. We observed that HSF1 overexpression remarkably increased the number of infiltrating α-SMA+ CAFs in tumor microenvironment, which was confirmed by IHC and western blot analyses using an anti-mouse α-SMA antibody (Fig. 7e-f). These findings revealed that HSF1 signaling in GBC is possibly associated with CAFs recruitment. To understand the molecular mechanisms by which HSF1 signaling promotes CAFs recruitment, we investigated the downstream effectors that may be responsible for the chemoattraction of CAFs. Through an RT-qPCR screen of a panel of factors responsible for CAFs recruitment [27], we observed that TGF-β1 and TGF-β2 were notably increased in GBC-SD cells overexpressing HSF1 and obviously decreased in NOZ cells with HSF1 depletion (Fig. 7g), findings that were further confirmed by western blot analysis (Fig. 7h). Consistently, the level of the phosphorylation of SMAD3 was also increased in GBC-SD xenograft tumors with enforced HSF1 expression (Fig. 7f). The findings of previous studies have demonstrated that TGF-β1 signaling is predominately responsible for maintaining myofibroblast/CAF phenotypes [28, 29], which can induce fibroblast activation and myofibroblast/CAF transdifferentiation. Thus, we further evaluated this signaling pathway with respect to fibroblast activation and CAF recruitment. Isolated peritumoral fibroblast (PTFs) were assessed for fibroblast activation after being cocultured with the CM of GBC cells. To determine whether paracrine TGF-β1 signaling plays a role in the mechanism by which HSF1 mediates fibroblast activation and the recruitment of infiltrating CAFs, TGF-β-neutralizing antibody and exogenous rh-TGF-β1 were used to modulate the concentration of TGF-β1 in the CM of GBC cells. Conditioned medium from HSF1-overexpressing GBC-SD cells profoundly induced PTF activation, which transdifferentiated into CAFs and exhibited enhanced migration, while these effects could be abrogated by treating the CM with the anti-TGF-β-neutralizing antibody (Additional file 10: Fig. S7 and Fig. 7i). In addition, when rh-TGF-β1 (5 ng/mL) was administered in conditioned medium from GBC-SD cells with control group, the activation and migratory capacity of PTFs was notably increased (Additional file 10: Fig. S7 and Fig. 7i). Collectively, these results indicate that TGF-β1 signaling is a major downstream chemoattractant of HSF1 signaling to promote fibroblast activation and CAFs recruitment.

### HSF1-induced paracrine signaling by TGFβ1 from GBC cells is the primary determinant for inducing TSP-4 expression and secretion in CAFs

**Fig. 8:**
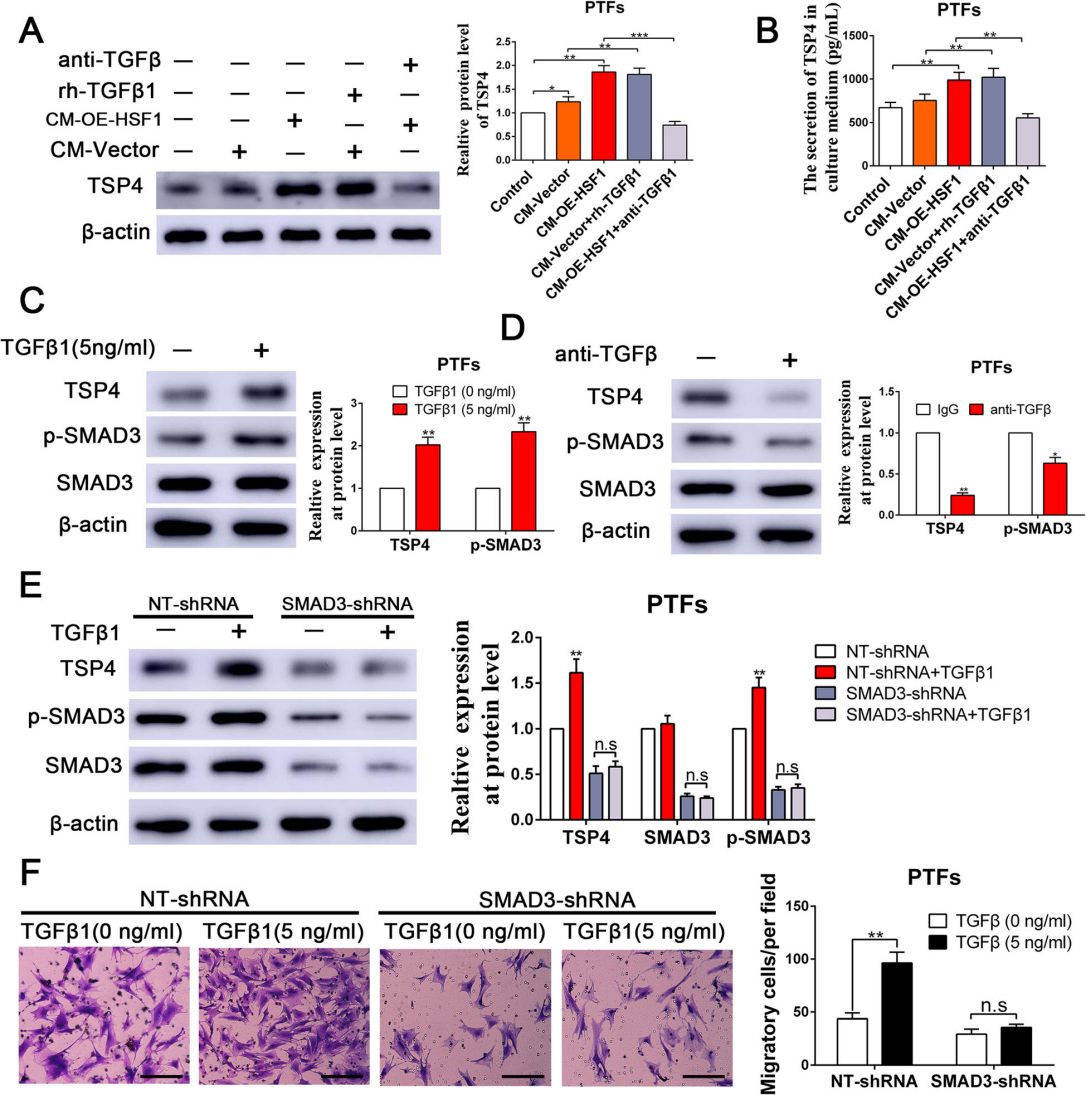
HSF1-mediated TGFβ signaling maintained the CAFs phenotypes and increased the expression and secretion of TSP-4. **a**, **b** The expression and secretion of TSP-4 with PTFs in response to CM-Vector, CM-OE-HSF1, CM-Vector+TGFβ1 or CM-OE-HSF1 + anti-TGFβ treatment was detected by western blot and Elisa assay respectively. n = three independent experiments, *P < 0.05 or **P < 0.01 by ANOVA. **c**, **d** TGFβ1 increased while TGFβ neutralizing antibody (anti-TGFβ) reduced the expression of TSP-4 and p-SMAD3 in PTFs. n = three independent experiments, *P < 0.05 or **P < 0.01 by Student’s t-test. **e** Knockdown of SMAD3 abrogated the TGFβ1-induced the expression of TSP-4. n = three independent experiments, **P < 0.01 by ANOVA. **f** Depletion of SMAD3 reversed the TGFβ1-induced the migration of PTFs. Scale bars = 50 μm. n = three independent experiments, **P < 0.01 by ANOVA

To further elucidate whether HSF1-mediated paracrine TGF-β signaling drives TSP-4 expression and secretion in CAFs, we collected CM from GBC-SD vector cells and GBC-SD OE-HSF1 cells, utilized TGF-β-neutralizing antibody and exogenous rh-TGF-β1 to modulate the concentration of TGF-β1, and then established an indirect coculture model to induce the transdifferentiation of PTFs into CAFs (Additional file 10: Fig. S7). Compared to the effects of CM from GBC-SD vector cells, treatment with CM from GBC-SD OE-HSF1 cells induced significant upregulation of TSP-4 expression and secretion in PTFs (Fig. 8a-b). Unsurprisingly, the anti-TGF-β-neutralizing antibody retarded CM from GBC-SD OE-HSF1 cells-induced upregulation of TSP-4 (Fig. 8a-b). Moreover, rh-TGF-β1 (5 ng/mL) was supplemented in CM of GBC-SD vector cells then incubated with PTFs, the expression level and secretion of TSP-4 was significantly enhanced (Fig. 8a-b). Next, we aspired to investigate the potential mechanism of TGF-β1-mediated TSP-4 upregulation in PTFs. We observed that rh-TGF-β1 fostered while anti-TGF-β neutralizing antibody mitigated the expression of TSP-4 and phosphorylation of SMAD3 (p-SMAD3), but the total SMAD3 level was unaltered (Fig. 8c-d). To assess whether TGF-β1 induces TSP-4 production via p-SMAD3, we transfected SMAD3 shRNA into PTFs to knockdown SMAD3 [19]. SMAD3 depletion curbed the increase levels of TSP-4 resulting from TGF-β1 stimulation of PTFs (Fig. 8e). Accordingly, rh-TGF-β1 could remarkably increase the migration of PTFs, while this promotion effect was abrogated by SMAD3 knockdown (Fig. 8f). These results also underscored that HSF1-induced paracrine of TGFβ from GBC cells is the primary determinant for the transdifferentiation of PTFs into CAFs and the subsequent upregulation of TSP-4 in CAFs.

## Discussion

GBC is characterized by a high rate of metastasis and recurrence, and the presence of cancer stem cells (CSCs) in GBC may explain both of these pathological properties. Intriguingly, the acquisition of cancer stem-like traits and EMT induction are closely associated [30]. EMT induction in mammary epithelial cells generates populations of cells that possess cancer stem-like traits with respect to their mammosphere formation, tumor-seeding capacity, and expression profiles for surface marker of stemness [30, 31]. In addition, CSCs are prone to exhibit enhancements for multiple attributes involved with mesenchymal transdifferentiation, such as increased vimentin and fibronectin expression and markedly increased expression of EMT-related transcription factors [30]. Furthermore, EMT and cancer stem-like features are modulated by a complex tumor microenvironmental network, and repression of EMT and cancer stem-like features is considered to be a promising strategy for the treatment of this highly refractory malignant tumor [32].

Recently, the tumor microenvironment has attracted a great deal of attention. Tumor initiation and progression have been well shown to be coevolutionary and reciprocal processes between tumor cells and the tumor microenvironment [33]. Through paracrine signaling, the disseminated cancer cells reeducate stromal fibroblasts and induce fibroblasts trans-differentiation into CAFs to generate a metastatic niche. CAFs then participate in the initiation and malignant progression of cancer [34, 35] by secreting chemokines or growth factors [36, 37], remodeling the ECM (extracellular matrix) [38], facilitating metastasis, repressing antitumor immune responses, fostering resistance to chemotherapy [7] and creating a niche for cancer stemness maintenance [10]. Consistently, our results showed that TSP-4, a novel stromal glycoprotein, promoted GBC cell proliferation, EMT and cancer stem-like properties in a paracrine manner. CAF-derived TSP-4 was shown to interact with the transmembrane receptor integrin α2 on GBC cells, which then activated Akt-mediated HSF1 activation to promote GSC cell proliferation and induce the EMT and cancer stem-like traits of gallbladder cancer. These findings underscore the importance of paracrine interactions between GBC cells and CAFs in tumor initiation and metastasis. Furthermore, activated HSF1 signaling further increased the expression and paracrine of TGF-β to induce the transdifferentiation of PTFs into CAFs, leading to their recruitment into GBC and increased TSP-4 expression in CAFs. We identified a positive feedback loop between GBC cells and CAFs, and targeting this reciprocal interaction loop may be a promising strategy for conquering gallbladder cancer.

**Fig. 9:**
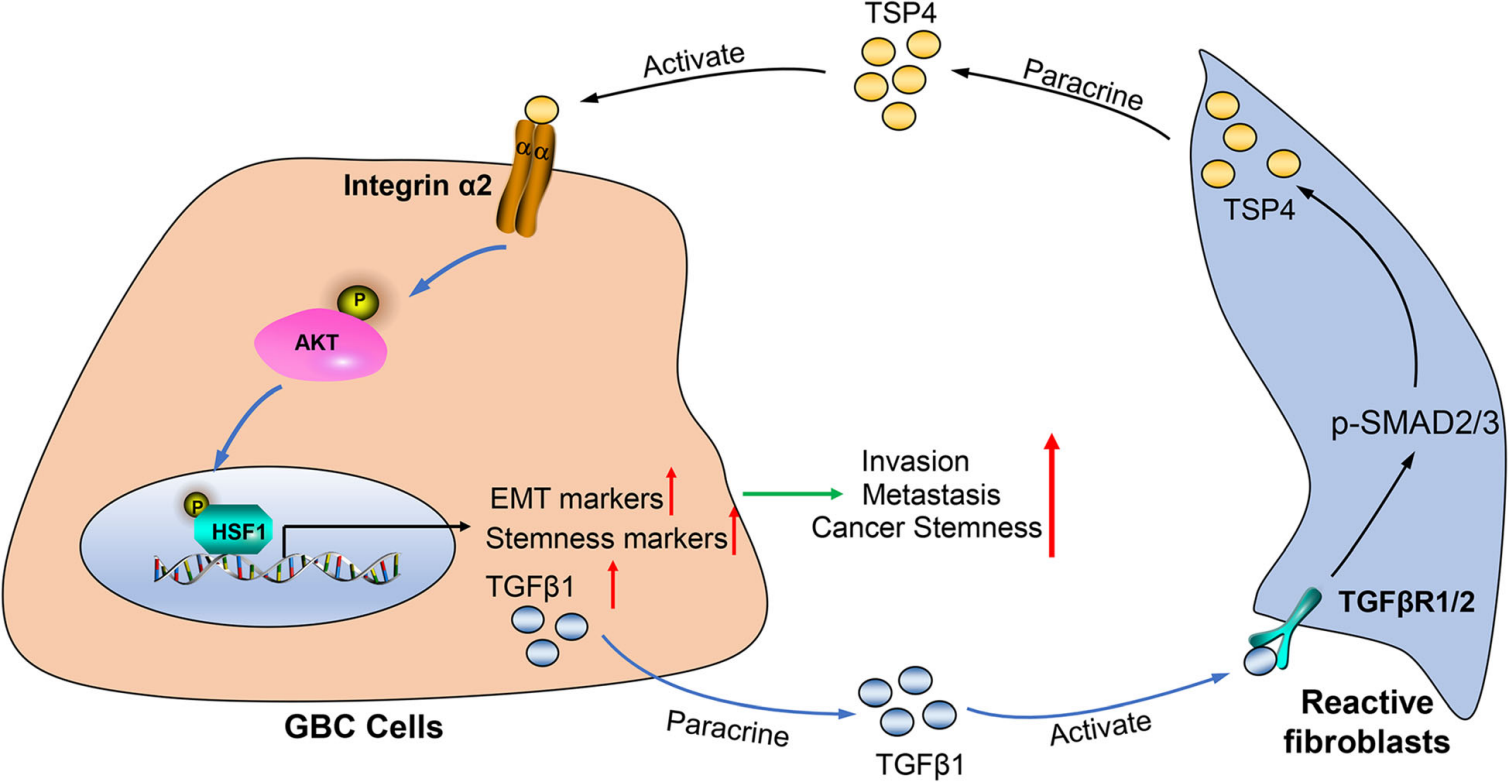
Schematic of the findings of the present study. The TSP-4 secreted by CAFs binds to integrin α2 on the surface of GBC cells, thus activating downstream signaling cascades, including upregulation the phosphorylation of AKT and HSF1(p-AKT: S473 and p-HSF1:S326), which induces nuclear translocation of p-HSF1, and triggers the EMT and cancer stem-like traits. Activated HSF1 signaling further increased the expression and paracrine of TGF-β to induce the transdifferentiation of reactive fibroblasts into CAFs, leading to their recruitment into GBC and increased TSP-4 expression in CAFs

The dysregulation of HSF1 signaling has been observed in a wide range of cancers [39–42], and this dysregulation is regarded as a pivotal regulator of EMT and cancer stem-like traits [22, 43]. Whole-exome and ultradeep sequencing of gallbladder cancer revealed a panel of genes with nonsilent mutations, including TP53, KRAS and ERBB signaling components (EGFR, ERBB2, ERBB3, and ERBB4 and their downstream genes) [1]. Intriguingly, TP53, EGFR and ERBB2 are all canonical upstream components of HSF1 signaling [44–46], suggesting that HSF1, as a downstream effector of these genes, is a potent enabler driving the malignant progression of GBC. Thus, we further explored whether the downstream transcription factor HSF1 participates in the TSP-4/integrin α2 axis-induced EMT and cancer stem-like traits in gallbladder cancer. In agreement with the aforementioned findings, we observed that HSF1 activation is essential for the CAFs-derived TSP-4-induced proliferation, EMT and cancer stemness of GBC cells. Moreover, our results further revealed that TSP-4/integrin α2 axis-induced Akt activation results in Akt phosphorylation of HSF-1 at S326, which leads to enhanced HSF-1 transcriptional activity. Collectively, these results led us to propose a model whereby CAFs-derived TSP-4 interacts with its receptor integrin α2 to activate the PI3K-Akt pathway, which results in HSF-1 activation that mediates the EMT and cancer stemness of gallbladder cancer (Fig. 9).

In the present, we also revealed that HSF1 signaling instigated communication between GBC cells and CAFs. By RT-qPCR screening a panel of factors responsible for CAFs recruitment, we showed that TGF-β1 and TGF-β2 expression could be notably influenced by manipulating HSF1 levels. Moreover, HSF1 was shown to activate TGF-β signaling to promote the recruitment of CAFs and stimulate SMAD3-mediated TSP-4 expression in a paracrine manner, thereby forming a positive feedback loop to drive the malignant progression of GBC (the proposed model is shown in Fig. 9). Our results are consistent with the findings of previous studies, demonstrating that TGF-β1 induces the upregulation of TSP-4 expression via activation of SMAD3 signaling to promote angiogenesis [22]. Intriguingly, previous investigations also disclosed that HSF1 can reprogram tumor stroma by modulating TGF-β and SDF1 signaling [29]. Taken together, our data indicate the complex TSP-4/integrin α2/HSF1/TGF-β cascade mediates reciprocal interactions between GBC cells and CAFs, providing a promising therapeutic target for gallbladder cancer patients.

The lack of specific symptoms at the early stage of GBC and the scarcity of biomarkers contribute to the poor diagnosis of GBC patients. Furthermore, due to the high rate of metastasis, the prognosis of GBC patients remains bleak [47, 48]. Thus, the identification of new biomarkers to promote the early diagnosis of GBC and uncovering their clinical prognosis value for GBC patients is urgently needed. Through double IF staining, we showed that HSF1 was primarily expressed in tumor cells and located in the nuclei of GBC cells, while abundant α-SMA+ CAFs surrounded HSF1+ GBC cells. Our IHC results also showed that positive TSP-4 staining was predominantly observed in stromal cells. Additionally, high TSP-4 expression in stromal cells was associated with poor clinical prognosis of GBC patients, indicating that TSP-4 may be considered as alternative diagnostic and prognostic biomarker in gallbladder cancer. Our data indicated that CAFs establish a supporting niche to maintain cancer stemness, and targeting the reciprocal interactions between GBC cells and CAFs may present a new strategy for conquering gallbladder cancer. However, CAFs are abundant, and heterogeneous populations of irreversibly activated fibroblasts are present in the tumor microenvironment comprising different subpopulations with distinct phenotypes and functions [7]. Thus, future investigations should focus on defining specific subsets of CAFs with unique roles in different GBC types, which will provide the crucial information needed for the development of precise therapies of GBC patients.

## Conclusions

In this study, through RT-qPCR screening the expression of thrombospondins, we found that TSP-4, as the stromal glycoprotein, was highly expressed in CAFs of GBC, and CAFs derived TSP-4 induced the proliferation, EMT and cancer stem-like features of GBC cells. Mechanistically, TSP-4 secreted from CAFs binds to the transmembrane receptor integrin α2 on GBC cells to induce the phosphorylation of HSF1 at S326 and maintained malignant phenotypes of GBC cells. Moreover, TSP-4/ integrin α2 axis-induced the phosphorylation of HSF1 at S326 is mediated by Akt activation (p-Akt at S473) in GBC cells. In addition, activated HSF1 signaling increased the expression and paracrine of TGF-β1 to induce the transdifferentiation of PTFs into CAFs, recruit them into GBC and foster TSP-4 expression in CAFs, thereby forming a positive feedback loop to drive the malignant progression of GBC. Our data indicate the complicated process of the TSP-4/ integrin α2/HSF1/TGF-β cascades-mediated the reciprocal interaction in GBC cells and CAFs, providing a promising therapeutic target for gallbladder cancer patients.

## Supplementary Information

**Additional file 1:** **Table S1.** A list of the utilized primary antibodies.

**Additional file 2: Table S2.** Primers sequences for real-time PCR analysis.

**Additional file 3: Table S3.** High expression of TSP4 in GBC stroma predicts poor prognosis.

**Additional file 4: Figure S1.** CAFs-derived TSP-4 promoted the proliferation of GBC cells. (A-D) GBC-SD and NOZ cells were incubated with CM-shVector, CM-shTSP-4, CM-shTSP-4 + rh-TSP-4, then the proliferation of GBC cells was determined by MTT, Colony formation and Edu assay respectively. The magnification of Edu picture is 200×. Scale bars = 50 μm. n = three independent experiments, *P < 0.05 or **P < 0.01 by ANOVA. (E) The ALDH+ cells populations in GBC-SD cells after CM-shVector, CM-shTSP-4, CM-shTSP-4 + rh-TSP-4 treatments were detected by Flow cytometric analysis. n = three independent experiments, *P < 0.05 or **P < 0.01 by ANOVA. (F) GBC-SD and NOZ cells were incubated with CM-shVector, CM-shTSP-4, CM-shTSP-4 + rh-TSP-4 for 48 h, the relative expression of stemness markers (CD44, Nanog, Oct4 and Sox2), and epithelial-mesenchymal transition markers (E-cadherin and vimentin) at protein level were analyzed and plotted. n = three independent experiments, *P < 0.05 or **P < 0.01 by ANOVA.

**Additional file 5: Figure S2.** rh-TSP-4 facilitated the proliferation, EMT and cancer stemness of GBC cells. (A) The knockdown efficacy of TSP-4 by transfection of TSP-4 shRNA into CAFs confirmed by western blot. n = three independent experiments, *P < 0.05 or **P < 0.01 by ANOVA. (B-D) The effects of rh-TSP-4 (25 nM) on the proliferation of GBC-SD and NOZ cells were assessed by MTT, Colony formation and Edu assay respectively. n = three independent experiments, *P < 0.05 or **P < 0.01 by Student’s t-test versus TSP-4 (0 nM) group (E) The ALDH+ cells populations in GBC-SD and NOZ cells after rh-TSP-4 (25 nM) treatments were detected by Flow cytometric analysis. n = three independent experiments, *P < 0.05 or **P < 0.01 by Student’s t-test. (F) GBC-SD and NOZ cells were incubated with rh-TSP-4 (25 nM) for 24 h, then the invasive ability of GBC cells was assessed by the Matrigel-invasion assay. The scale bars = 50 μm. n = three independent experiments, **P < 0.01 by Student’s t-test. (G) Representative images of the tumorsphere formation assay after rh-TSP-4 (25 nM) treatments in GBC-SD and NOZ cells. The number of tumorspheres was counted and plotted, and the percentage of tumorspheres with diameters of 50–100 μm, 100–150 μm or > 150 μm was calculated and plotted. The scale bar represents 50 μm. Magnification is × 200, and scale bars = 50 μm. n = three independent experiments, **P < 0.01 by Student’s t-test. (H) The expression of EMT and CSC markers after rh-TSP-4 (0, 10, 25 and 50 nM) treatments were evaluated by western blotting analysis. β-Actin was used as an internal control. n = three independent experiments, *P < 0.05 or **P < 0.01 by ANOVA versus TSP-4 (0 nM).

**Additional file 6: Figure S3.** Integrin α2 mediates the effects of paracrine of TSP-4 signaling on the proliferation of GBC cells. (A-C) GBC-SD and NOZ cells were incubated with rh-TSP-4, rh-TSP-4 + anti-α2 or anti-α2, then the proliferation of GBC cells was determined by MTT, Colony formation and Edu assay respectively. n = three independent experiments, *P < 0.05 or **P < 0.01 by ANOVA. (D) GBC-SD and NOZ cells were incubated with rh-TSP-4, rh-TSP-4 + anti-α2 or anti-α2 for 48 h, the relative expression of stemness markers (CD44, Nanog, Oct4 and Sox2), and epithelial-mesenchymal transition markers (E-cadherin and vimentin) at protein level were analyzed and plotted. β-Actin was used as an internal control. n = three independent experiments, *P < 0.05, **P < 0.01 or # *P* < 0.01 by ANOVA versus control group.

**Additional file 7: Figure S4.** HSF1 activation plays a vital role on rh-TSP-4 induced proliferation of GBC cells. (A) GBC-SD and NOZ cells were treated with rh-TSP-4, rh-TSP-4 + anti-α2 or anti-α2 for 48 h, the relative expression of p-HSF1, HSP90 and HSP70 at protein level were analyzed and plotted. β-Actin was used as an internal control. n = three independent experiments, *P < 0.05, **P < 0.01 or # *P* < 0.01 by ANOVA versus control group. (B-D) GBC-SD and NOZ cells were treated with rh-TSP-4, si-HSF1or rh-TSP-4 + si-HSF1, then the proliferation of GBC cells was determined by MTT, Colony formation and Edu assay respectively. n = three independent experiments, *P < 0.05 or **P < 0.01 by ANOVA. (E) GBC-SD and NOZ cells were incubated with rh-TSP-4, si-HSF1or rh-TSP-4 + si-HSF1, then the relative expression of stemness markers (CD44, Nanog, Oct4 and Sox2), and epithelial-mesenchymal transition markers (E-cadherin and vimentin) at protein level were analyzed and plotted. β-Actin was used as an internal control. n = three independent experiments, *P < 0.05, **P < 0.01 or # *P* < 0.01 by ANOVA versus control group.

**Additional file 8: Figure S5.** Blocking AKT signaling abrogated the TSP-4/integrin α2 axis induced the EMT and cancer stemness of GBC cells. (A) Representative images of the Matrigel invasion assay after rh-TSP-4, rh-TSP-4 + LY294002 or LY294002 treatments in GBC-SD and NOZ cells. Scale bars = 50 μm. n = three independent experiments, **P < 0.01 or ^#^ *P* < 0.01by ANOVA versus control group. (B) Representative images of the tumorsphere formation assay after rh-TSP-4, rh-TSP-4 + LY294002 or LY294002 treatments in GBC-SD and NOZ cells. The number of tumorspheres was counted and plotted, and the percentage of tumorspheres with diameters of 50–100 μm, 100–150 μm or > 150 μm was calculated and plotted. The scale bar represents 50 μm. Magnification is × 200, and scale bars = 50 μm. n = three independent experiments, **P < 0.01 or ^#^ *P* < 0.01 by ANOVA versus control group. (C) The expression of EMT and CSC markers (E-cadherin, Vimentin, CD44, Nanog, Oct4 and Sox2) after rh-TSP-4, rh-TSP-4 + LY294002 or LY294002 treatments were evaluated by western blotting. (D) The ALDH+ cells populations after rh-TSP-4, rh-TSP-4 + LY294002 or LY294002 treatments were detected by Flow cytometric analysis. n = three independent experiments, *P < 0.05 or **P < 0.01 or # *P* < 0.01by ANOVA versus control group.

**Additional file 9: Figure S6.** Inhibition of AKT signaling reversed the rh-TSP-4 induced proliferation of GBC cells. (A-C) GBC-SD and NOZ cells were treated with rh-TSP-4, rh-TSP-4 + LY294002 or LY294002, then the proliferation of GBC cells was determined by MTT, Colony formation and Edu assay respectively. n = three independent experiments, *P < 0.05, **P < 0.01 or # *P* < 0.01 by ANOVA versus control group. (D) GBC-SD and NOZ cells were incubated with rh-TSP-4, rh-TSP-4 + LY294002 or LY294002, then the relative expression of stemness markers (CD44, Nanog, Oct4 and Sox2), and epithelial-mesenchymal transition markers (E-cadherin and vimentin) at protein level were analyzed and plotted. β-Actin was used as an internal control. n = three independent experiments, *P < 0.05, **P < 0.01 or # *P* < 0.01 by ANOVA versus control group.

**Additional file 10: Figure S7.** HSF1-mediated TGFβ1 paracrine signaling in GBC induced PTFs activation and transdifferentiated into CAFs. (A) The PTFs were incubated with CM-Vector, CM-OE-HSF1, CM-Vector+TGFβ1 or CM-OE-HSF1 + anti-TGFβ, then RT-qPCR was conducted to show the expression of CAFs markers: α-SMA, fibronectin and Col 1α in PTFs. n = three independent experiments, *P < 0.05, or **P < 0.01 by ANOVA versus control group. (B) IF staining of α-SMA displayed that CM-OE-HSF1 or CM-Vector+TGFβ1 induced PTFs activation and transdifferentiated into CAFs, while TGFβ neutralizing antibody reversed the CM-OE-HSF1 induced PTFs activation. The magnification of the picture is 400×. Scale bars = 20 μm.

## Data Availability

All data generated or analyzed during this study are included either in this article or in the supplementary information files.

## Abbreviations

CAFs: Cancer-associated fibroblasts
GBC: Gallbladder cancer
THBSs or TSPs: Thrombospondins
PTFs: Peritumoral fibroblast
EMT: Epithelial–mesenchymal transition
HSF1: Heat shock factor1
TGF-β: Transforming growth factor β
IGF-II: Insulin-like growth factor-II
IGF1R: Insulin-like growth factor 1 receptor
IL-1α/β: Interleukin-1α/β
CXCL9: Chemokine (C-X-C motif) ligand 9
CXCL10: Chemokine (C-X-C motif) ligand 10
CXCR3: Chemokine (C-X-C motif) receptor 3
COMP: Cartilage oligomeric matrix protein
CD36: Cluster of differentiation 36
CM: Conditioned medium
EdU: Ethynyl deoxyuridine
ELISA: The enzyme-linked immunosorbent assay
Co-IP: Coimmunoprecipitation
ALDH: Alcohol dehydrogenase
IHC: Immunohistochemical staining
α-SMA: Alpha smooth muscle actin
Sox2: (sex determining region Y)-box 2
CD44: Cluster of differentiation 44
Oct4: (octamer-binding transcription factor 4)
SMAD3: Mother against decapentaplegic homolog 3
CSCs: Cancer stem cells
EGFR: Epidermal growth factor receptor
SDF1: Stromal cell-derived factor-1
