# Supplementary material for: Thrombospondin 4/integrin α2/HSF1 axis promotes proliferation and cancer stem-like traits of gallbladder cancer by enhancing reciprocal crosstalk between cancer-associated fibroblasts and tumor cells
Source: J Exp Clin Cancer Res. 2021 Jan 6;40:14. doi: 10.1186/s13046-020-01812-7 (PMC7789630; doi:10.1186/s13046-020-01812-7)
Supplement: Supplementary file 1 — Additional file 1: Table S1. A list of the utilized primary antibodies. [file 13046_2020_1812_MOESM1_ESM.docx]

**Additional file 1: Table S1.** A list of the utilized primary antibodies

| **Antibody** | **Dilution & Use** | **Company** |
| --- | --- | --- |
| Rabbit anti-E-cadherin | 1:1000 (WB) | Cell Signaling Technology |
| Goat anti-TSP-4 | 1:1000 (WB) | R&D Systems |
|  | 1:400 (IHC) |  |
| Rabbit anti-vimentin | 1:1000 (WB) | Cell Signaling Technology |
| Rabbit anti-CD44 | 1:1000 (WB) | Abcam |
|  | 1:300 (IF) |  |
| Rabbit anti-Sox2 | 1:1000 (WB) | Abcam |
| Rabbit anti-Oct4 | 1:1000 (WB) | Abcam |
| Rabbit anti-Nanog | 1:1000 (WB) | Abcam |
| Mouse anti- Integrin α2 | 20µg/ml(neutralization) | Millpore |
| Rabbit anti-ki-67 | 1:500 (IHC) | Abcam |
| Rabbit anti-HSF1 | 1:2000 (WB) | Abcam |
| Rabbit anti-p-HSF1(S326) | 1:1000 (WB) | Abcam |
| Rabbit anti-HSP90 | 1:1000 (WB) | Abcam |
| Rabbit anti-HSP70 | 1:1000 (WB) | Abcam |
| Rabbit anti-AKT | 1:1000 (WB) | Cell Signaling Technology |
| Rabbit anti-p-AKT(S473) | 1:1000 (WB) | Cell Signaling Technology |
| Mouse anti-p-ERK | 1:1000 (WB) | Cell Signaling Technology |
| Rabbit anti-ERK | 1:1000 (WB) | Cell Signaling Technology |
| Rabbit anti-p-JNK | 1:1000 (WB) | Cell Signaling Technology |
| Mouse anti-JNK | 1:1000 (WB) | Cell Signaling Technology |
| Rabbit anti-p-mTOR | 1:1000 (WB) | Cell Signaling Technology |
| Rabbit anti-mOTR | 1:1000 (WB) | Cell Signaling Technology |
| Rabbit anti-p-p38 | 1:1000 (WB) | Cell Signaling Technology |
| Rabbit anti-p38 | 1:1000 (WB) | Cell Signaling Technology |
| Rabbit anti-p-SMAD3 | 1:1000 (WB) | Cell Signaling Technology |
| Rabbit anti-SMAD3 | 1:1000 (WB) | Cell Signaling Technology |
| Rabbit anti-Col I | 1:1000 (WB) | Abcam |
| Rabbit anti Fibronectin | 1:1000 (WB) | Abcam |
| Rabbit anti-α-SMA | 1:500 (WB);1:200 (IF) | Abcam (ab5694) |
| Rabbit anti-α-SMA (mice) | 1:1000 (WB) | Abcam (ab32575) |
| Rabbit anti-TGFβ1 | 1:500 (WB) | Abcam |
| Rabbit anti-TGFβ2 | 1:500 (WB) | Abcam |
| Rabbit anti-TGFβ | 4µg/ml (neutralization) | R&D system |
| Goat anti-rabbit IgG-HRP | 1:10,000 (WB) | Abbkine. Inc |
| Goat anti-mouse IgG-HRP | 1:10,000 (WB) | Abbkine. Inc |
| Goat anti-Rabbit dylight 594  (red) IgG antibody | 1:150 (IF) | Abbkine. Inc |
