# Supplementary material for: Thrombospondin 4/integrin α2/HSF1 axis promotes proliferation and cancer stem-like traits of gallbladder cancer by enhancing reciprocal crosstalk between cancer-associated fibroblasts and tumor cells
Source: J Exp Clin Cancer Res. 2021 Jan 6;40:14. doi: 10.1186/s13046-020-01812-7 (PMC7789630; doi:10.1186/s13046-020-01812-7)
Supplement: Supplementary file 2 — Additional file 2: Table S2. Primers sequences for real-time PCR analysis. [file 13046_2020_1812_MOESM2_ESM.docx]

**Additional file 2: Table S2.** Primers sequences for real-time PCR analysis

| **Gene** | **Primer Sequences** |
| --- | --- |
| α-SMA | Forward: 5′-GATGGTGGGAATGGGACAAA-3′ |
|  | Reverse: 5′-GCCATGTTCTATCGGGTACTTC-3′ |
| TGFβ3 | Forward: 5′-GCTGTCTGCCCTAAAGGAATTA-3′  Reverse: 5′-CCGGAATTCTGCTCGGAATAG-3′ |
| IL-1A | Forward: 5′-TGTGACTGCCCAAGATGAAG-3′  Reverse: 5′-CGTGAGTTTCCCAGAAGAAGAG-3′ |
| TGFβ1 | Forward: 5′-CGTGGAGCTGTACCAGAAATAC-3′  Reverse: 5′-CACAACTCCGGTGACATCAA-3′ |
| Fibronectin | Forward: 5′-CCACAGTGGAGTATGTGGTTAG-3′ |
|  | Reverse: 5′-CAGTCCTTTAGGGCGATCAAT-3′ |
| CXCL-1 | Forward: 5′-CGAAGTCATAGCCACACTCAA-3′ |
|  | Reverse: 5′-GATTTGTCACTGTTCAGCATCTT-3′ |
| CXCL-2 | Forward: 5′-TCACCTCAAGAACATCCAAAGT-3′ |
|  | Reverse: 5′-CAAGCTTTCTGCCCATTCTTG-3′ |
| CXCL-3 | Forward: 5′-TCACCTCAAGAACATCCAAAGT-3′ |
|  | Reverse: 5′-AGACAAGCTTTCTTCCCATTCT-3′ |
| CXCL-5 | Forward: 5′-TCTGCAAGTGTTCGCCATAG-3′ |
|  | Reverse: 5′-GGGCTTCTGGATCAAGACAAA-3′ |
| CXCL-8 | Forward: 5′-CTTGGCAGCCTTCCTGATTT-3′ |
|  | Reverse: 5′-GGGTGGAAAGGTTTGGAGTATG-3′ |
| CCL-2 | Forward: 5′-TCATAGCAGCCACCTTCATTC-3′ |
|  | Reverse: 5′-CTCTGCACTGAGATCTTCCTATTG-3′ |
| CCL-5 | Forward: 5′-GAAATGGGTTCGGGAGTACAT-3′ |
|  | Reverse: 5′-AGGACAAGAGCAAGCAGAAA-3′ |
| TGFβ2 | Forward: 5′-GGATGCGGCCTATTGCTTTA-3′  Reverse: 5′-GTACCCTTTGGGTTCGTGTATC-3′ |
| Col 1α | Forward: 5′-CTAAAGGCGAACCTGGTGAT-3′  Reverse: 5′-TCCAGGAGCACCAACATTAC-3′ |
| TSP1 | Forward: 5′-CTGGACTCGCTGTAGGTTATG-3′ |
|  | Reverse: 5′-AGCATAGTCATCGTCCCTTTC-3′ |
| PDGF-D | Forward: 5′-GAAATTGTGGCTGTGGAACTG-3′ |
|  | Reverse: 5′-GGCCAGGCTCAAACTGTAATA-3′ |
| IL-6 | Forward: 5′-GGAGACTTGCCTGGTGAAA-3′  Reverse: 5′-CTGGCTTGTTCCTCACTACTC-3′ |
| WNT7A | Forward: 5′-CGTTCACCTACGCCATCATT-3′  Reverse: 5′-GTACTGGCCTTGCTTCTCTTT-3′ |
| IL-1B | Forward: 5′-ATGGACAAGCTGAGGAAGATG-3′  Reverse: 5′-CCCATGTGTCGAAGAAGATAGG-3′ |
| TSP-2 | Forward: 5′-GGACTTCAGTGGCACATTCT-3′  Reverse: 5′-TGCTTCCACATCACCACATAG-3′ |
| TSP-3 | Forward: 5′-GGACTTGGAGATGAGTGTGATG-3′  Reverse: 5′-GAGTCCTTCTGATTGGGATTGG-3′ |
| TSP-4 | Forward: 5′-CCTTCTGAGACAGCAGGTTAAG-3′  Reverse: 5′-GTCGGAGACTGAAACTTGAGAG-3′ |
| TSP-5 | Forward: 5′-GACAGTGATGGCGATGGTATAG-3′  Reverse: 5′-TCACAAGCATCTCCCACAAA-3′ |
| GAPDH | Forward: 5′-ACCACAGTCCATGCCATCAC-3′ |
|  | Reverse: 5′-TCCACCACCCTGTTGCTGAT-3′ |
