# Supplementary material for: Thrombospondin 4/integrin α2/HSF1 axis promotes proliferation and cancer stem-like traits of gallbladder cancer by enhancing reciprocal crosstalk between cancer-associated fibroblasts and tumor cells
Source: J Exp Clin Cancer Res. 2021 Jan 6;40:14. doi: 10.1186/s13046-020-01812-7 (PMC7789630; doi:10.1186/s13046-020-01812-7)
Supplement: Supplementary file 3 — Additional file 3: Table S3. High expression of TSP4 in GBC stroma predicts poor prognosis. [file 13046_2020_1812_MOESM3_ESM.docx]

| Characteristics |  | Cases  (N=75) | Number of patients | | *P* Value |
| --- | --- | --- | --- | --- | --- |
|  |  |  | TSP4^high in stroma^  (n=38) | TSP4^low in stroma^  (n=37) |  |
| Ages(years) | <60 | 42 | 22 | 20 | 0.941 |
|  | ≥60 | 33 | 17 | 16 |  |
| Gender | Male | 22 | 9 | 13 | 0.276 |
|  | Female | 53 | 29 | 24 |  |
| Tumor size (cm) | <3 | 45 | 17 | 28 | **0.006** |
|  | ≥3 | 30 | 21 | 9 |  |
| Histology | Well/moderate | 48 | 25 | 23 | 0.744 |
| differentiation | Poor | 27 | 13 | 14 |  |
| Lymph node | Present | 34 | 27 | 7 | **0.001** |
| metastasis | Absent | 31 | 11 | 30 |  |
| TNM stage | Ⅰ+Ⅱ | 16 | 9 | 7 | 0.615 |
| (AJCC) | Ⅲ+Ⅳ | 59 | 29 | 30 |  |
