# Supplementary figures and images for: Thrombospondin 4/integrin α2/HSF1 axis promotes proliferation and cancer stem-like traits of gallbladder cancer by enhancing reciprocal crosstalk between cancer-associated fibroblasts and tumor cells
Source: J Exp Clin Cancer Res. 2021 Jan 6;40:14. doi: 10.1186/s13046-020-01812-7 (PMC7789630; doi:10.1186/s13046-020-01812-7)

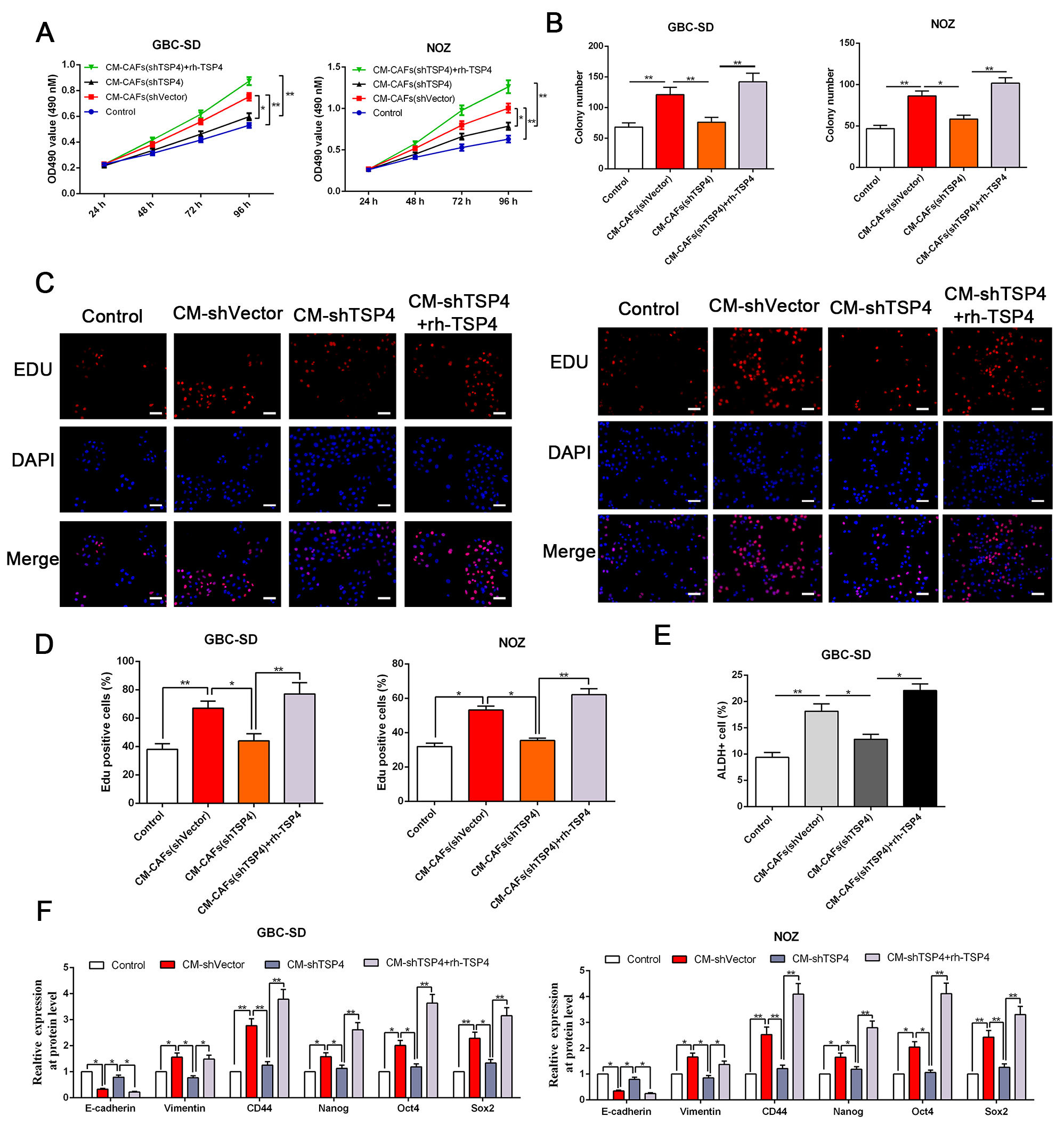

Supplement: Supplementary file 4 — Additional file 4: Figure S1. CAFs-derived TSP-4 promoted the proliferation of GBC cells. (A-D) GBC-SD and NOZ cells were incubated with CM-shVector, CM-shTSP-4, CM-shTSP-4 + rh-TSP-4, then the proliferation of GBC cells was determined by MTT, Colony formation and Edu assay respectively. The magnification of Edu picture is 200×. Scale bars = 50 μm. n = three independent experiments, *P < 0.05 or **P < 0.01 by ANOVA. (E) The ALDH+ cells populations in GBC-SD cells after CM-shVector, CM-shTSP-4, CM-shTSP-4 + rh-TSP-4 treatments were detected by Flow cytometric analysis. n = three independent experiments, *P < 0.05 or **P < 0.01 by ANOVA. (F) GBC-SD and NOZ cells were incubated with CM-shVector, CM-shTSP-4, CM-shTSP-4 + rh-TSP-4 for 48 h, the relative expression of stemness markers (CD44, Nanog, Oct4 and Sox2), and epithelial-mesenchymal transition markers (E-cadherin and vimentin) at protein level were analyzed and plotted. n = three independent experiments, *P < 0.05 or **P < 0.01 by ANOVA. [file 13046_2020_1812_MOESM4_ESM.tif]

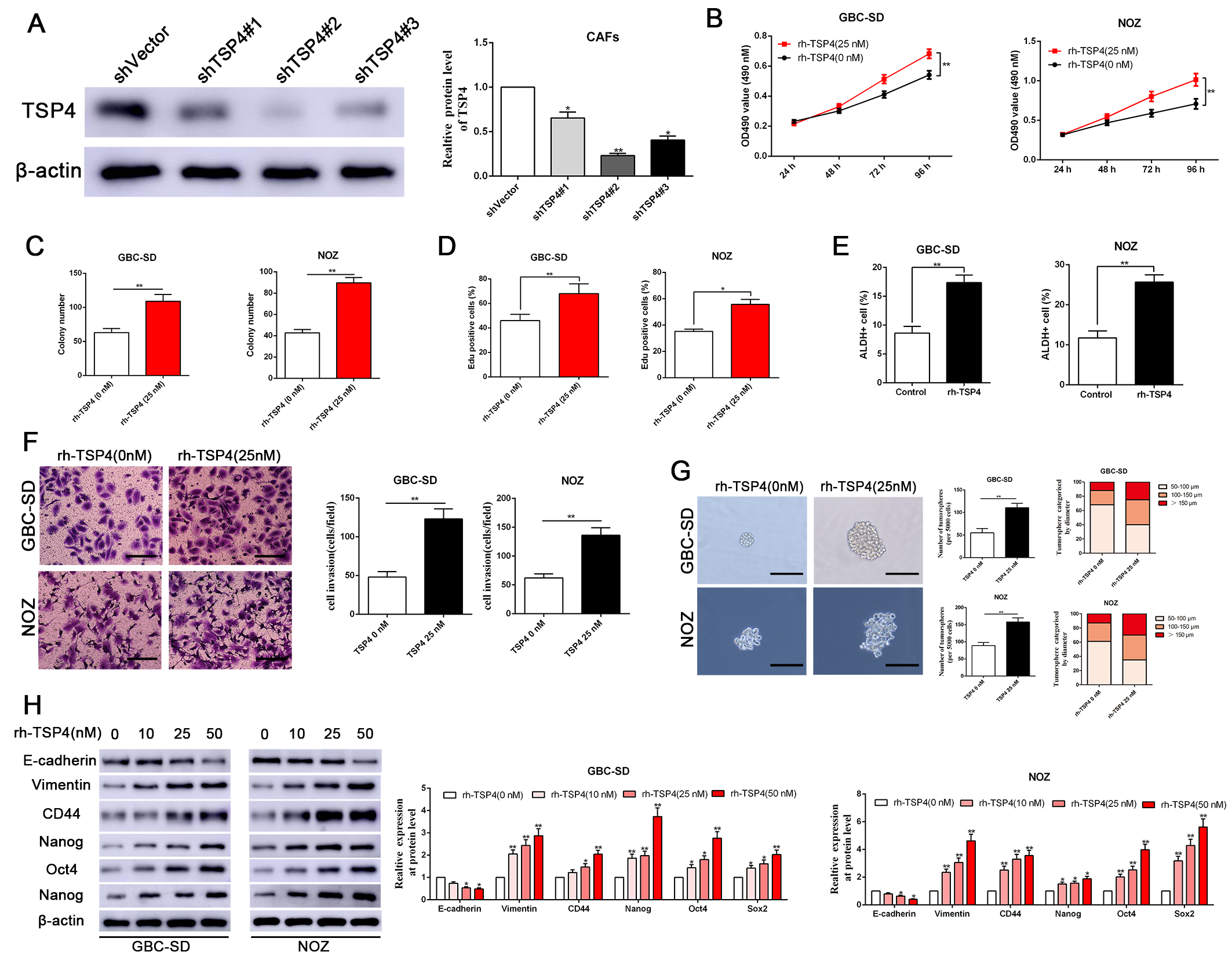

Supplement: Supplementary file 5 — Additional file 5: Figure S2. rh-TSP-4 facilitated the proliferation, EMT and cancer stemness of GBC cells. (A) The knockdown efficacy of TSP-4 by transfection of TSP-4 shRNA into CAFs confirmed by western blot. n = three independent experiments, *P < 0.05 or **P < 0.01 by ANOVA. (B-D) The effects of rh-TSP-4 (25 nM) on the proliferation of GBC-SD and NOZ cells were assessed by MTT, Colony formation and Edu assay respectively. n = three independent experiments, *P < 0.05 or **P < 0.01 by Student’s t-test versus TSP-4 (0 nM) group (E) The ALDH+ cells populations in GBC-SD and NOZ cells after rh-TSP-4 (25 nM) treatments were detected by Flow cytometric analysis. n = three independent experiments, *P < 0.05 or **P < 0.01 by Student’s t-test. (F) GBC-SD and NOZ cells were incubated with rh-TSP-4 (25 nM) for 24 h, then the invasive ability of GBC cells was assessed by the Matrigel-invasion assay. The scale bars = 50 μm. n = three independent experiments, **P < 0.01 by Student’s t-test. (G) Representative images of the tumorsphere formation assay after rh-TSP-4 (25 nM) treatments in GBC-SD and NOZ cells. The number of tumorspheres was counted and plotted, and the percentage of tumorspheres with diameters of 50–100 μm, 100–150 μm or > 150 μm was calculated and plotted. The scale bar represents 50 μm. Magnification is × 200, and scale bars = 50 μm. n = three independent experiments, **P < 0.01 by Student’s t-test. (H) The expression of EMT and CSC markers after rh-TSP-4 (0, 10, 25 and 50 nM) treatments were evaluated by western blotting analysis. β-Actin was used as an internal control. n = three independent experiments, *P < 0.05 or **P < 0.01 by ANOVA versus TSP-4 (0 nM). [file 13046_2020_1812_MOESM5_ESM.tif]

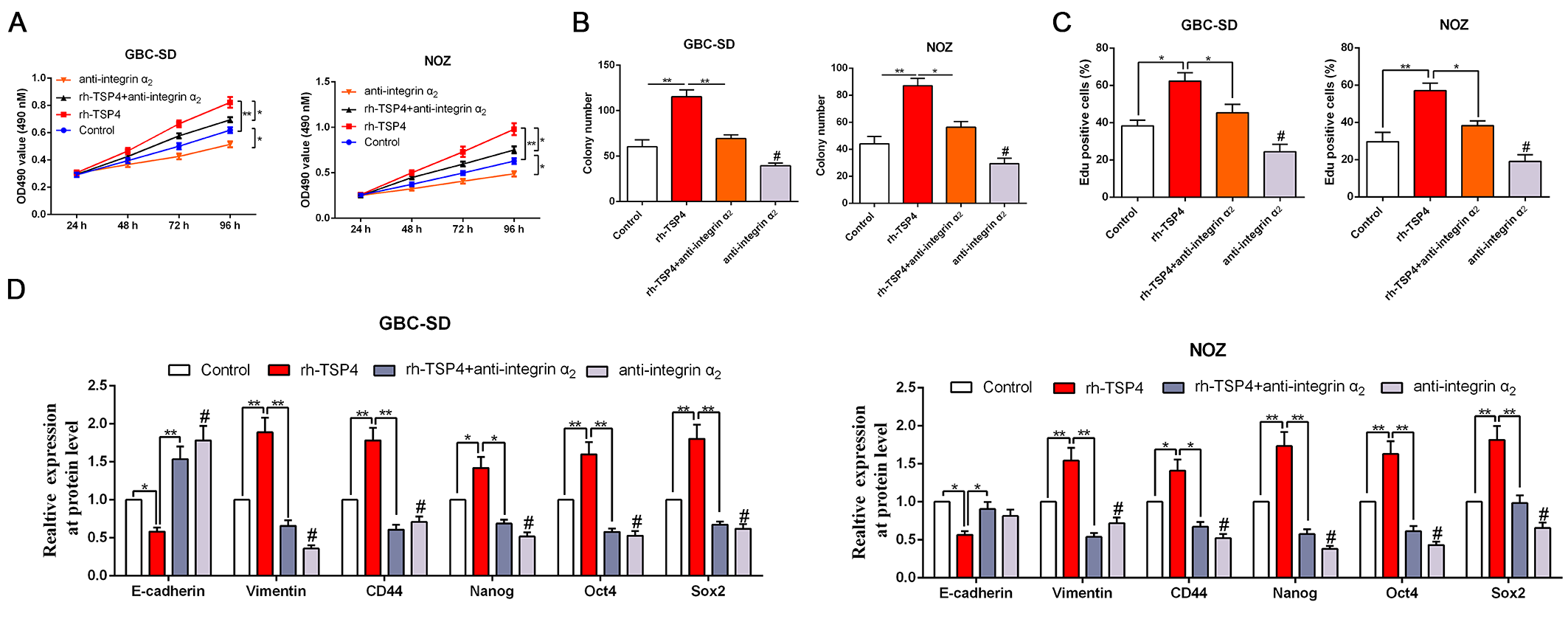

Supplement: Supplementary file 6 — Additional file 6: Figure S3. Integrin α2 mediates the effects of paracrine of TSP-4 signaling on the proliferation of GBC cells. (A-C) GBC-SD and NOZ cells were incubated with rh-TSP-4, rh-TSP-4 + anti-α2 or anti-α2, then the proliferation of GBC cells was determined by MTT, Colony formation and Edu assay respectively. n = three independent experiments, *P < 0.05 or **P < 0.01 by ANOVA. (D) GBC-SD and NOZ cells were incubated with rh-TSP-4, rh-TSP-4 + anti-α2 or anti-α2 for 48 h, the relative expression of stemness markers (CD44, Nanog, Oct4 and Sox2), and epithelial-mesenchymal transition markers (E-cadherin and vimentin) at protein level were analyzed and plotted. β-Actin was used as an internal control. n = three independent experiments, *P < 0.05, **P < 0.01 or # P < 0.01 by ANOVA versus control group. [file 13046_2020_1812_MOESM6_ESM.tif]

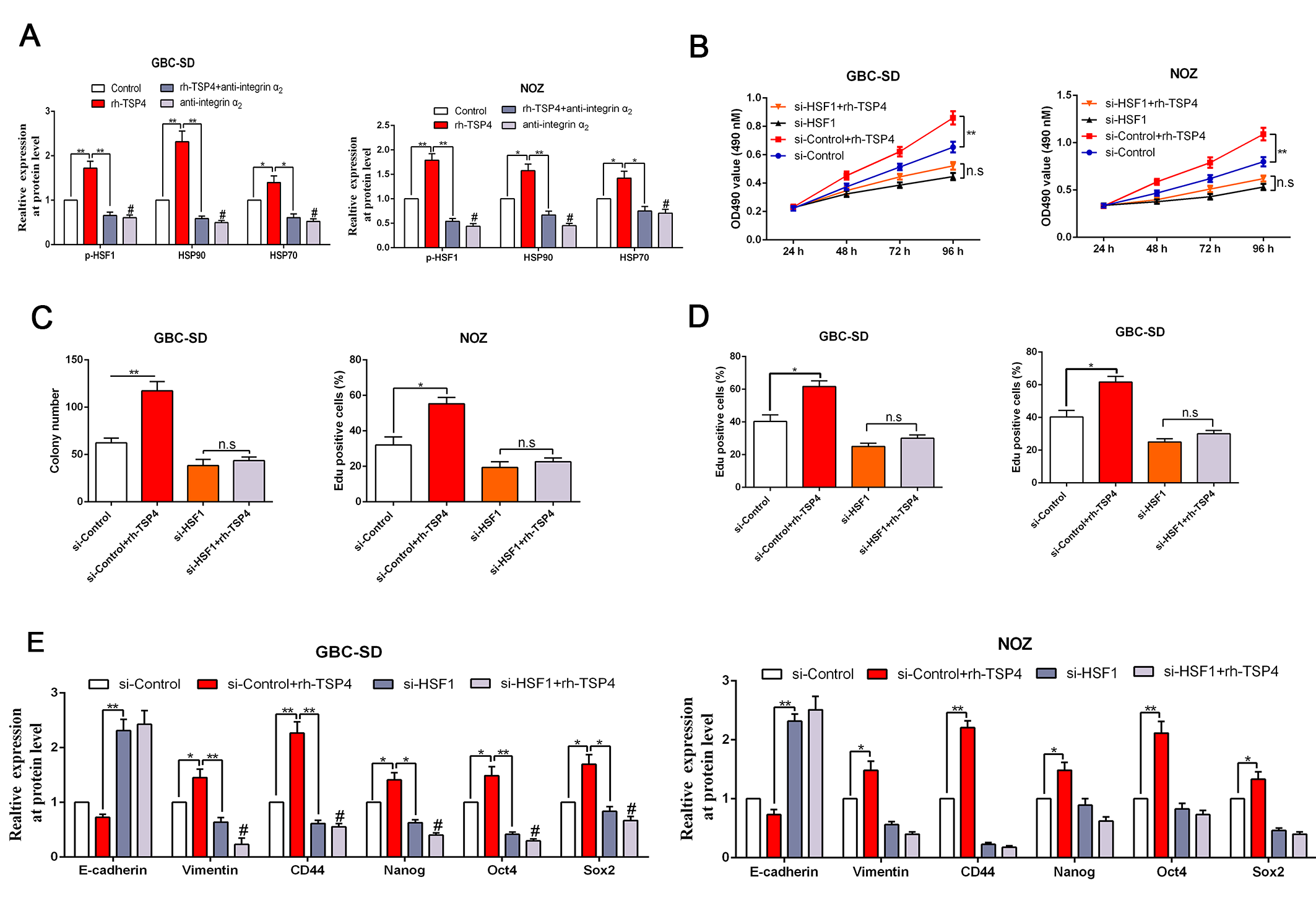

Supplement: Supplementary file 7 — Additional file 7: Figure S4. HSF1 activation plays a vital role on rh-TSP-4 induced proliferation of GBC cells. (A) GBC-SD and NOZ cells were treated with rh-TSP-4, rh-TSP-4 + anti-α2 or anti-α2 for 48 h, the relative expression of p-HSF1, HSP90 and HSP70 at protein level were analyzed and plotted. β-Actin was used as an internal control. n = three independent experiments, *P < 0.05, **P < 0.01 or # P < 0.01 by ANOVA versus control group. (B-D) GBC-SD and NOZ cells were treated with rh-TSP-4, si-HSF1or rh-TSP-4 + si-HSF1, then the proliferation of GBC cells was determined by MTT, Colony formation and Edu assay respectively. n = three independent experiments, *P < 0.05 or **P < 0.01 by ANOVA. (E) GBC-SD and NOZ cells were incubated with rh-TSP-4, si-HSF1or rh-TSP-4 + si-HSF1, then the relative expression of stemness markers (CD44, Nanog, Oct4 and Sox2), and epithelial-mesenchymal transition markers (E-cadherin and vimentin) at protein level were analyzed and plotted. β-Actin was used as an internal control. n = three independent experiments, *P < 0.05, **P < 0.01 or # P < 0.01 by ANOVA versus control group. [file 13046_2020_1812_MOESM7_ESM.tif]

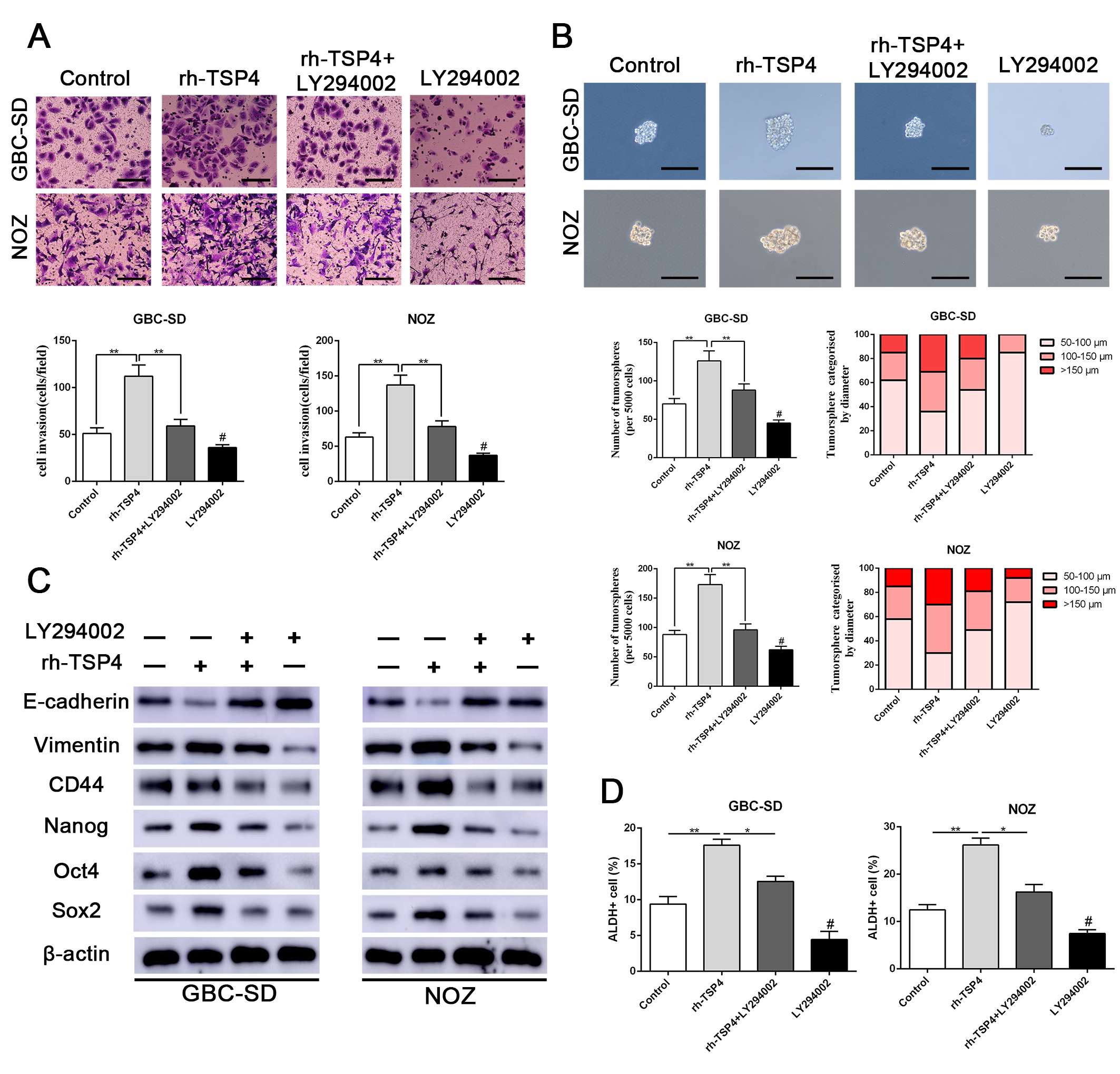

Supplement: Supplementary file 8 — Additional file 8: Figure S5. Blocking AKT signaling abrogated the TSP-4/integrin α2 axis induced the EMT and cancer stemness of GBC cells. (A) Representative images of the Matrigel invasion assay after rh-TSP-4, rh-TSP-4 + LY294002 or LY294002 treatments in GBC-SD and NOZ cells. Scale bars = 50 μm. n = three independent experiments, **P < 0.01 or # P < 0.01by ANOVA versus control group. (B) Representative images of the tumorsphere formation assay after rh-TSP-4, rh-TSP-4 + LY294002 or LY294002 treatments in GBC-SD and NOZ cells. The number of tumorspheres was counted and plotted, and the percentage of tumorspheres with diameters of 50–100 μm, 100–150 μm or > 150 μm was calculated and plotted. The scale bar represents 50 μm. Magnification is × 200, and scale bars = 50 μm. n = three independent experiments, **P < 0.01 or # P < 0.01 by ANOVA versus control group. (C) The expression of EMT and CSC markers (E-cadherin, Vimentin, CD44, Nanog, Oct4 and Sox2) after rh-TSP-4, rh-TSP-4 + LY294002 or LY294002 treatments were evaluated by western blotting. (D) The ALDH+ cells populations after rh-TSP-4, rh-TSP-4 + LY294002 or LY294002 treatments were detected by Flow cytometric analysis. n = three independent experiments, *P < 0.05 or **P < 0.01 or # P < 0.01by ANOVA versus control group. [file 13046_2020_1812_MOESM8_ESM.tif]

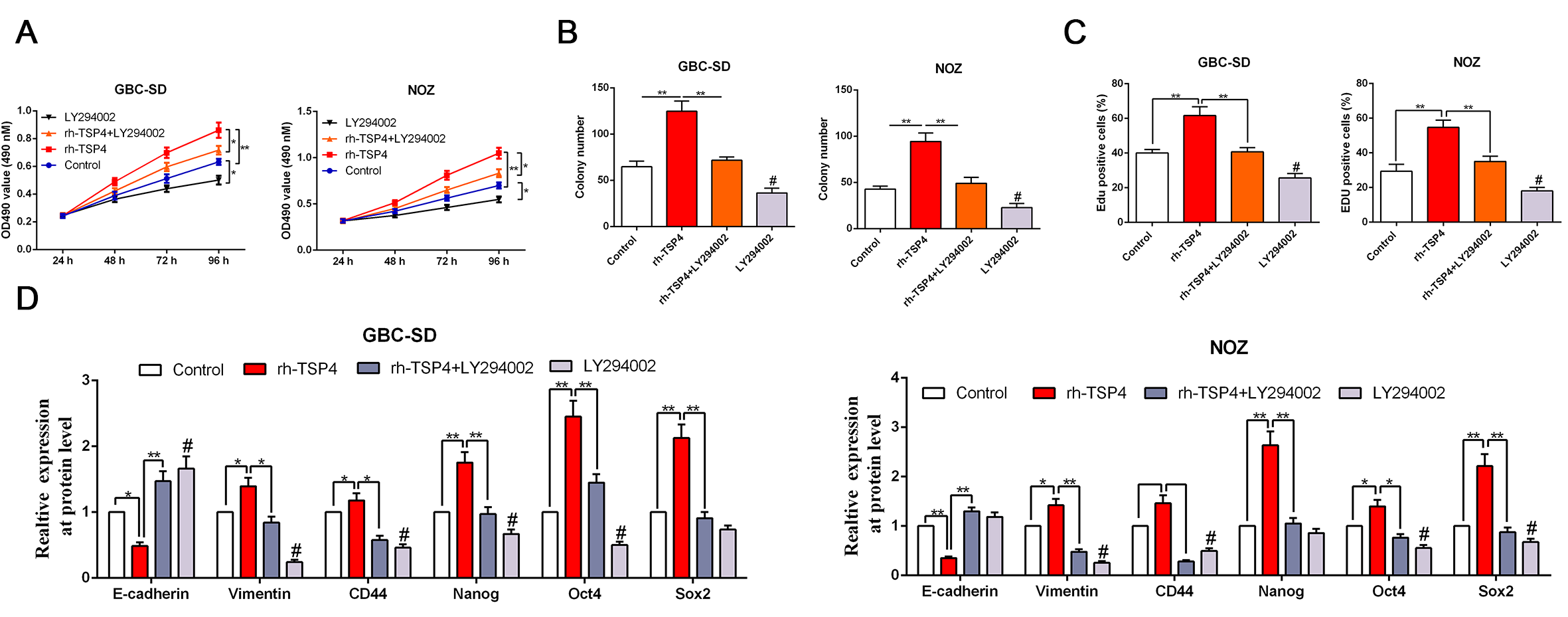

Supplement: Supplementary file 9 — Additional file 9: Figure S6. Inhibition of AKT signaling reversed the rh-TSP-4 induced proliferation of GBC cells. (A-C) GBC-SD and NOZ cells were treated with rh-TSP-4, rh-TSP-4 + LY294002 or LY294002, then the proliferation of GBC cells was determined by MTT, Colony formation and Edu assay respectively. n = three independent experiments, *P < 0.05, **P < 0.01 or # P < 0.01 by ANOVA versus control group. (D) GBC-SD and NOZ cells were incubated with rh-TSP-4, rh-TSP-4 + LY294002 or LY294002, then the relative expression of stemness markers (CD44, Nanog, Oct4 and Sox2), and epithelial-mesenchymal transition markers (E-cadherin and vimentin) at protein level were analyzed and plotted. β-Actin was used as an internal control. n = three independent experiments, *P < 0.05, **P < 0.01 or # P < 0.01 by ANOVA versus control group. [file 13046_2020_1812_MOESM9_ESM.tif]

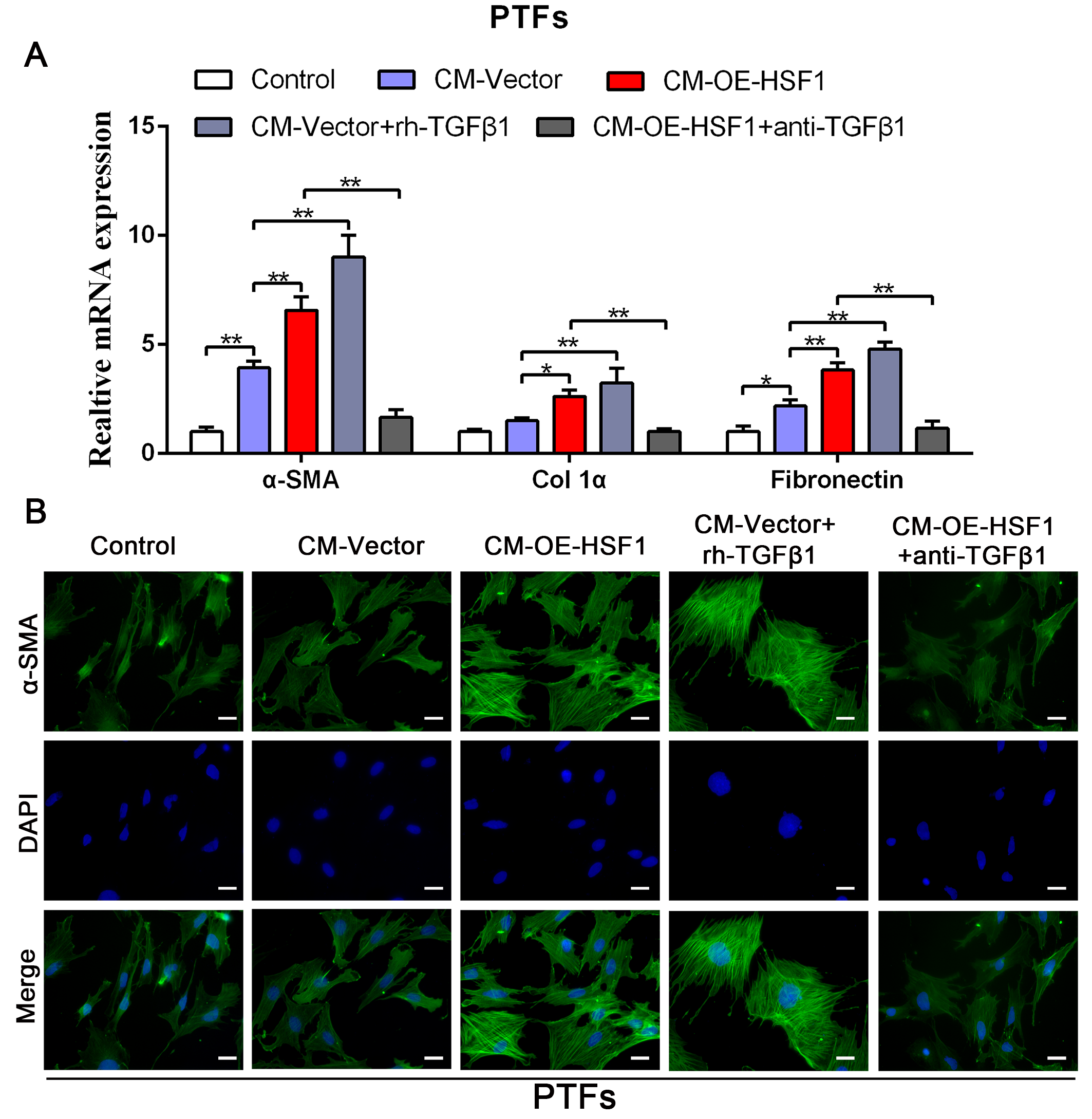

Supplement: Supplementary file 10 — Additional file 10: Figure S7. HSF1-mediated TGFβ1 paracrine signaling in GBC induced PTFs activation and transdifferentiated into CAFs. (A) The PTFs were incubated with CM-Vector, CM-OE-HSF1, CM-Vector+TGFβ1 or CM-OE-HSF1 + anti-TGFβ, then RT-qPCR was conducted to show the expression of CAFs markers: α-SMA, fibronectin and Col 1α in PTFs. n = three independent experiments, *P < 0.05, or **P < 0.01 by ANOVA versus control group. (B) IF staining of α-SMA displayed that CM-OE-HSF1 or CM-Vector+TGFβ1 induced PTFs activation and transdifferentiated into CAFs, while TGFβ neutralizing antibody reversed the CM-OE-HSF1 induced PTFs activation. The magnification of the picture is 400×. Scale bars = 20 μm. [file 13046_2020_1812_MOESM10_ESM.tif]
